# Operational assessment tool for forest carbon dynamics for the United States: a new spatially explicit approach linking the LUCAS and CBM-CFS3 models

**DOI:** 10.1186/s13021-022-00201-1

**Published:** 2022-02-02

**Authors:** Benjamin M. Sleeter, Leonardo Frid, Bronwyn Rayfield, Colin Daniel, Zhiliang Zhu, David C. Marvin

**Affiliations:** 1 U.S. Geological Survey, Seattle, WA USA; 2 Apex Resource Management Solutions Ltd., Ottawa, ON Canada; 3 U.S. Geological Survey, Reston, VA USA; 4 Salo Sciences Inc, San Francisco, CA USA

**Keywords:** Land use, Climate change, Carbon balance, CBM-CFS3, LUCAS

## Abstract

**Background:**

Quantifying the carbon balance of forested ecosystems has been the subject of intense study involving the development of numerous methodological approaches. Forest inventories, processes-based biogeochemical models, and inversion methods have all been used to estimate the contribution of U.S. forests to the global terrestrial carbon sink. However, estimates have ranged widely, largely based on the approach used, and no single system is appropriate for operational carbon quantification and forecasting. We present estimates obtained using a new spatially explicit modeling framework utilizing a “gain–loss” approach, by linking the LUCAS model of land-use and land-cover change with the Carbon Budget Model of the Canadian Forest Sector (CBM-CFS3).

**Results:**

We estimated forest ecosystems in the conterminous United States stored 52.0 Pg C across all pools. Between 2001 and 2020, carbon storage increased by 2.4 Pg C at an annualized rate of 126 Tg C year^−1^. Our results broadly agree with other studies using a variety of other methods to estimate the forest carbon sink. Climate variability and change was the primary driver of annual variability in the size of the net carbon sink, while land-use and land-cover change and disturbance were the primary drivers of the magnitude, reducing annual sink strength by 39%. Projections of carbon change under climate scenarios for the western U.S. find diverging estimates of carbon balance depending on the scenario. Under a moderate emissions scenario we estimated a 38% increase in the net sink of carbon, while under a high emissions scenario we estimated a reversal from a net sink to net source.

**Conclusions:**

The new approach provides a fully coupled modeling framework capable of producing spatially explicit estimates of carbon stocks and fluxes under a range of historical and/or future socioeconomic, climate, and land management futures.

## Background

Forest ecosystems play a critical role in the global carbon cycle. Annually, the global forest sink has been estimated at ~1.1 Pg C year^−1^ [1], with more recent studies placing the sink as high as 2.0 Pg C year^−1^ [2], although uncertainties in those estimates are substantial. This net sink currently represents 25–50% of the global C emissions due to fossil fuels [1, 3], highlighting the magnitude and importance the world’s forests play in the global carbon cycle. The Intergovernmental Panel on Climate Change (IPCC) estimates that globally, forests have the capacity to absorb 25% of the atmospheric CO_2_ needed under a scenario limiting global warming to 2 °C [4]. Regionally, temperate forests of North America have been estimated as a large and persistent carbon sink at a rate of 0.3–0.9 Pg C year^−1^ [5], with U.S. forests accounting for ~80% of this net carbon uptake [6]. The large contribution of U.S. forests has been attributed primarily to the growth of immature forests over the past several decades, as a result of the recovery of forests from harvest and other disturbances combined with the creation of new forests due to conversions from other land cover types. However, uncertainties in the size and future direction of this forest-based sink remain, due in large part to limitations regarding the methodological approaches used to estimate the net carbon flux [5].

Given their importance in the global carbon cycle, U.S. forests are increasingly looked to as a potential means of offsetting greenhouse-gas emissions from fossil fuel consumption and land use. The implementation of natural climate solutions, representing portfolios of land management policies and actions aimed at increasing sequestration of ecosystem carbon, provides an opportunity to make land management decisions today that will serve to maintain, and possibly increase, this key carbon sink into the future [7–9]. Such decisions, however, require robust methods and tools in order to meaningfully quantify, compare and contrast the projected response of U.S. carbon stocks and fluxes to these alternative land management strategies.

Various approaches have been used to estimate past and future carbon stocks and fluxes in U.S. forests, including process-based ecosystem models, inventory-based “stock-change” methods, and carbon budget “stock-flow” models [10, 11]. For example, process-based ecosystem models are designed to represent underlying biogeochemical processes, including a wide range of carbon dynamics [12–15]; such models are typically used to simulate vegetation responses to a range of controlling processes and applied at global to national scales. A key strength of this class of model is its ability to integrate the findings from manipulative experiments into projections of the effects of major controlling processes on carbon dynamics. Ecosystem models can also readily be applied across large geographic areas, and can be used to integrate and extend experimental understanding in order to generate projections under novel future conditions [16]. Future projections generated by ecosystem models generally come with high uncertainty, however, as these models often require a large

number of input parameters, many of which can be difficult to estimate over large spatial extents [5, 16]. Furthermore, ecosystem models are typically limited in their ability to represent variation in forest types, often relying on generalizations to compensate for unknown parameters; for example, vegetation communities are often generalized into a small set of plant functional types, rather than retaining the specific forest types or species groups recorded in forest inventories. Finally, ecosystem models generally lack the ability to integrate the effects of complex socioeconomic processes, such as land conversions, into their projections of future ecosystem carbon dynamics [12, 17], thus limiting their ability to project the consequences of alternative land management scenarios.

An alternative to process-based ecosystem models for estimating forest carbon stocks and fluxes are inventory- based “stock-change” approaches, where field-based measurements are used directly to estimate changes in forest carbon stocks between two successive time periods [10]. For example, using the U.S. Department of Agriculture Forest Inventory and Analysis (FIA) data, estimates of changes in carbon stocks are produced annually for U.S. ecosystems and reported to the United Nations Framework Convention on Climate [18]. FIA data provide a comprehensive set of plot-level data characterizing forest attributes and have been used in a number of national studies aimed at quantifying the U.S. forest carbon sink [5, 19–22]. However, in the context of assessing the carbon consequences of land management alternatives, there are several limitations associated with inventory-based stock-change accounting, including: (1) assessments across large geographic extents can be cost-prohibitive due to the need for repeated plot measurements across a large number of sample sites in order to fully characterize ecosystem heterogeneity; (2) inventory-based approaches are not spatially explicit and thus often rely on inaccurate extrapolation methods to develop estimates in areas where no inventory exists [23]; (3) stock-change methods do not capture the underlying drivers of carbon fluxes that are typically required to separate the effects of land management alternatives in future projections; and (4) stock-change methods do not readily allow for projections under novel conditions (i.e., those outside of historical plot conditions), such as those that are expected to occur in the future under alternative global change scenarios. However, when applied in conjunction with other models, a robust forest inventory program can be used to calibrate and validate the parameters for other models, allowing model projections to be more readily extended across larger spatial extents and novel conditions [11, 17].

The third approach to estimating forest carbon stocks and fluxes is to use a stock-flow (also called “gain-loss” or “carbon budget”) approach. Here the total ecosystem carbon is divided into a number of carbon pools, with the model then explicitly tracking the fluxes of carbon between pools over time [10, 11, 24]. With stock-flow models, flux rates between pools are typically derived from inventory data. By tracking the forest type and age of each simulated entity (e.g., stand or cohort), empirically derived regional stem wood “volume curves,” representing the relationship between stem wood biomass and forest type/age, provide the foundation for calculating annual flux rates due to growth. Biomass expansion factors are then used to convert stem wood growth rates to fluxes in other biomass pools, such as roots, snags and branches [10]. More complex stock-flow models also track the explicit dynamics of dead organic matter (DOM) pools, including soil carbon dynamics, based on empirically derived measurements of DOM decay and decomposition rates.

A widely used stock-flow model is the CBM-CFS3, a carbon budget model originally developed for use in Canada [11]. The CBM-CFS3 tracks the annual carbon fluxes between 21 different pools (10 biomass and 11 DOM), including fluxes to wood products and to/from the atmosphere. Importantly, fluxes due to disturbances (e.g., fire, harvest, land conversion) are also considered, as are changes in flux rates over time to variation in temperature (e.g., due to climate change), making the approach well suited for forecasting the consequences of future land management scenarios on forest carbon. Thanks to its general and transparent structure, all of CBM-CFS3 input parameters can be customized to reflect regional conditions; as a result it has been applied in many different countries [25–28], including studies within the U.S. [29, 30]. A current limitation of the CBM-CFS3, however, is that it generates only spatially referenced (rather than spatially explicit) projections – i.e., the model currently tracks carbon by landscape strata, rather than by spatial cells, and thus no spatial interactions between strata are possible. As a result the model is unable to account for complex spatial patterns of land cover change (e.g., urbanization, fire spread), such as those typically found in operational scenarios of land management; it is also not able to assign carbon values to specific locations (i.e., cells) across a landscape.

To overcome some of these limitations in projecting the carbon consequences of future land management scenarios, we previously developed the Land Use and Carbon Simulator (LUCAS) [31]. LUCAS is a modeling framework capable of providing spatially explicit estimates of carbon stocks and fluxes in response to alternative future land management scenarios, and consists of two integrated components: a state-and- transition simulation model (STSM) of land use/land cover (LULC) change, combined with a stock-flow model of carbon dynamics [32, 33]. The STSM portion of LUCAS projects the consequences of disturbances and land management actions (i.e., land cover conversions) across a landscape, with uncertainty, while the stock-flow carbon model then infers the carbon consequences of these changes. While LUCAS has already been applied in the U.S. at local [34], state [31], and national [35] scales, in both spatially explicit and spatially referenced forms, to date the model’s carbon budget flux rates have been derived from repeated simulations of the Integrated Biosphere Simulator (IBIS), a process-based ecosystem model [17, 35]. When used with LUCAS, output from a suite of IBIS simulations is transformed into age and ecosystem-dependent carbon growth and turnover rates, providing the flux rates required for the LUCAS stock-flow carbon model.

Like most process-based ecosystem models, however, IBIS is both: (1) limited to a relatively small number of discrete vegetation classes (plant functional types), which in turn limit its ability to represent the full heterogeneity of forested ecosystems across the U.S. and (2) computationally intensive, limiting its ability to run in a spatially explicit manner across larger landscapes.

In this paper we present the results of our efforts to improve our existing LUCAS framework by adding the rich suite of carbon dynamics embodied within the existing CBM-CFS3, creating an operational forecasting tool capable of rapidly generating spatially explicit estimates of historical and future carbon stocks and fluxes, under alternative future land management scenarios, for any forested location in the conterminous U.S. (CONUS). We estimate historical (2001–2020) carbon stocks and flux and compare this to other CONUS-scale efforts to quantify carbon. To probe the sensitivity of the major controlling processes (climate, land-use land-cover change (LULC), disturbance) on ecosystem carbon, we ran additional simulations with these processes removed and compared the results to our historical reference scenario. We then demonstrate the ability of the model to extend annual projections into the future (2021–2050), based on climate change scenarios. Two regional case studies are also presented: first, an estimation of historical annual wildfire emissions for the state of California and, second, simulations of the effect of regional-scale reforestation on the carbon balance of forested ecosystems in the western U.S.

## Results

The integrated modeling approach described in “Methods” produces estimates of the composition of landscape classes and their attributes (e.g., age, time-since-transition), landscape transitions, carbon stocks, and carbon fluxes. For each variable type, the model can output both spatially explicit (i.e., raster maps) and spatially referenced estimates. Spatially referenced results are provided for each of the three stratification systems used in the model (ecoregions, states, and ownership). We also show how the approach can be used to develop future projections and to provide near real-time assessments of carbon fluxes from disturbances. We use net biome productivity (NBP) to reflect the net carbon sink in ecosystems after impacts from land use and disturbance are accounted for. We follow the convention where positive NBP values indicate a net sink of carbon in terrestrial ecosystems while negative values indicate a net source to the atmosphere.

### Comparison of historical carbon stocks and flux estimates

#### Estimation of carbon storage in live, dead, and soil pools

Using the linked LUCAS-CBM modeling approach, we estimated total ecosystem carbon storage (TEC; the sum of all carbon stored in live, DOM, and soil pools) in forested ecosystems of CONUS. In 2001, forest TEC was estimated at 52,027 Tg C. Live carbon accounted for 31% (16,201 Tg C) of TEC while DOM pools accounted for 69% (35,826 Tg C). Within DOM pools, standing and down deadwood accounted for 5% (2731 Tg C), litter accounted for 4% (2239 Tg C), and soil organic carbon accounted for 59% (30,856 Tg C) of TEC.

**Fig. 1:**
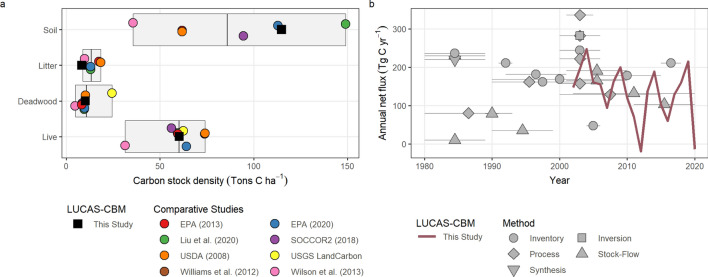
Comparison of estimates of forest carbon stocks and net flux with other studies. Panel **a** shows estimates of carbon stock density. Bars represent the range of values in other studies with the median shown as the black vertical bar. Panel **b** shows estimates of annual net biome productivity. Point and line ranges represent the reported net carbon sink and the time period reported in other studies using various methods. The red line shows the annual estimates from this study. Note, inventory methods reflect a range of studies, which include use of both FIA and US EPA greenhouse-gas inventory data

Our estimates of total carbon storage compare well with other recent studies. For example, in the most recent national greenhouse gas inventory, the U.S. Environmental Protection Agency (EPA) estimated forested ecosystems stored 17,940 Tg C in live biomass [18], slightly higher than our estimate of 16,201 Tg C (mean estimated over the 2001–2010 period). When normalizing for differences in forest area (Fig. 1), EPA estimated carbon storage in live vegetation was 64.1 tons C ha^−1^, compared to our estimate of 60.3 tons C ha^−1^. Other carbon pool estimates also compared well with those from the EPA. We estimated carbon stored in dead biomass averaged 10.2 tons C ha^−1^ compared to EPA’s estimate of 9.8 tons C ha^−1^, while soil carbon storage was estimated at 114.8 tons C ha^−1^ compared to EPA’s estimate of 112.8 tons C ha^−1^. Our estimates of litter carbon density (8.3 tons C ha^−1^) were toward to lower range of other studies and comparable to estimates from [36] (9.7 tons C ha^−1^) and [6] (8.8 tons C ha^−1^), while the EPA estimated litter carbon density at 13 tons C ha^−1^.

#### Net change in ecosystem carbon storage

**Fig. 2:**
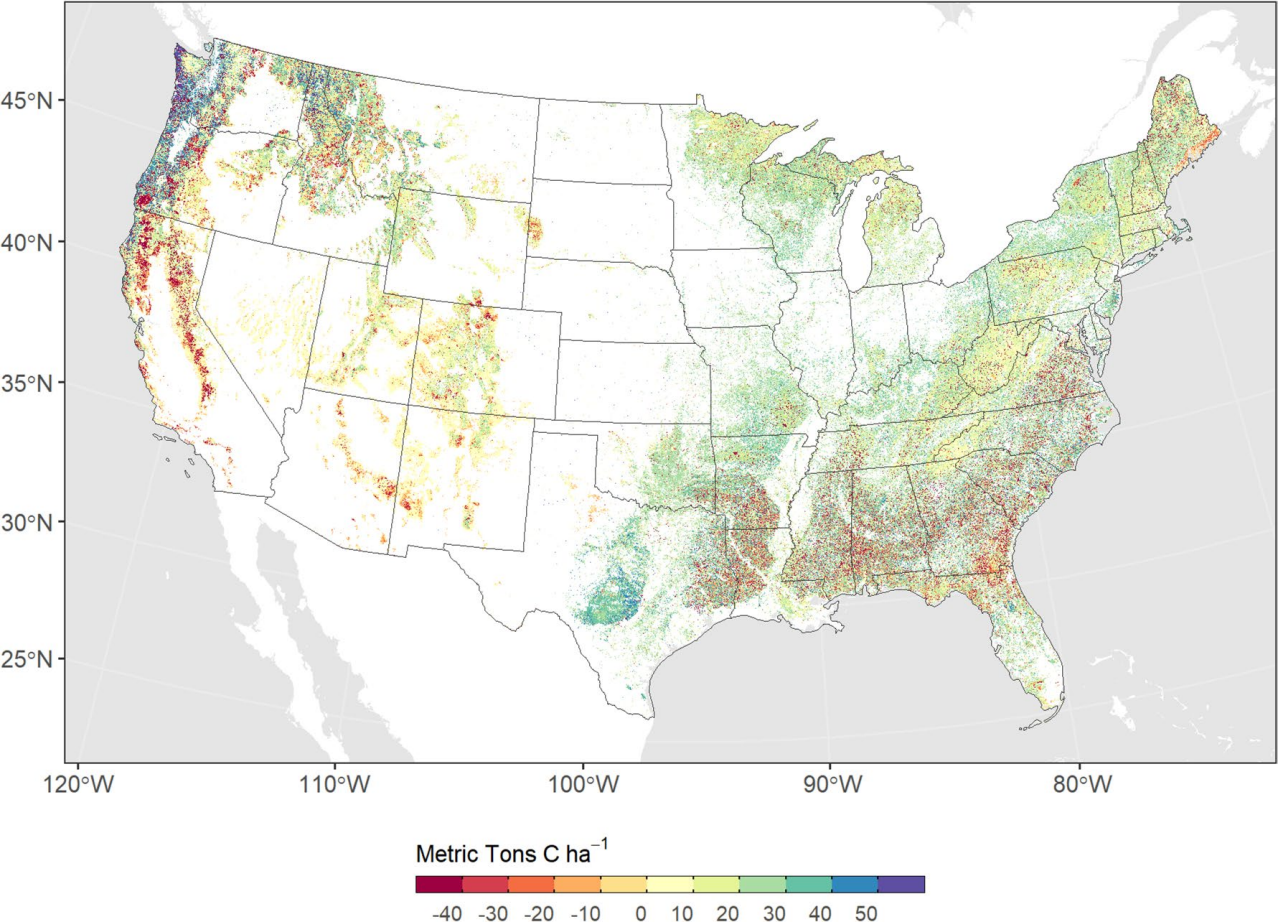
Map of the total estimated net biome productivity of conterminous U.S. forests for the period 2001–2020. Negative values indicate a net loss of carbon from ecosystems and positive values indicate a net sink of carbon. Very high negative NBP values are the result of disturbance losses, such as those resulting from harvesting and wildfire

Between 2001 and 2020, TEC increased from 52,027 Tg C to 54,413 Tg C, resulting in a forest carbon sink of 2386 Tg C over the 19-year period. On an annualized basis, the net forest carbon sink was estimated to be 126 Tg C year^−1^, or 0.47 Tons C ha^−1^ year^−1^. We estimated large variability in the size of the forest carbon sink over time, primarily due to the effects of weather and climate variability and its resulting effect on net primary production (NPP); variability in the rate of disturbance also played an important role, especially in the later part of the study period. For the 2001–2010 period we estimated the net forest sink to be 165 Tg C year^−1^ compared to 103 Tg C year^−1^ for the 2011–2020 period (Fig. 1). On an annual basis, NBP ranged from a sink of 247 Tg C year^−1^ to a net source at a rate of −19 Tg C year^−1^. Figure 2 shows the spatial distribution of NBP in U.S. forests. Forests in California and the Southeast were the largest drivers of carbon loss, with fire driving a majority of the change in the west and forest harvest driving changes in the Southeast. The largest sinks of carbon were located in the coastal Pacific Northwest and in redwood forests of California. In general, the largest carbon sink rates were located in young regenerating stands while older stands had smaller sink rates or were carbon neutral.

Comparison of net carbon flux estimates between models and approaches can be difficult due to differences in boundary conditions, ecosystem processes included in models (e.g., effects of land use), and the temporal period covered. In general, our estimates of NBP are within the range of numerous other studies which generally range from a low of 47 Tg C year^−1^ [37] to a high of 282 Tg C year^−1^ [5]. [5] found that on average, atmospheric inversion (282 Tg C year^−1^) and inventory-based methods (244 Tg C year^−1^) produced higher estimates of the net forest carbon sink than estimates derived using terrestrial biosphere models (158 Tg C year^−1^). However, all approaches showed large uncertainties. The Second State of the Carbon Cycle Report estimated the U.S. forest carbon sink at 178 Tg C year^−1^ (0.58 tons C ha^−1^ year^−1^) for the period 2000–2014 [6]. Over a similar period (2001–2014), the LUCAS-CBM approach estimated a net sink rate of 0.53 tons C ha^−1^ year^−1^. Using a process-based dynamic global vegetation model, [17] estimated the net forest sink at 125 Tg C year^−1^ while over the same period we estimated the net sink at 165 Tg C year^−1^, which was similar to the mean estimate of terrestrial biosphere models evaluated by [5]. Ref. [5] reviewed 17 terrestrial biosphere models and found a mean net ecosystem exchange (NEE) in U.S. forests of −157.6 (SD = 309.5) Tg C year^−1^ (here, negative values denote a net sink of carbon in ecosystems and product pools). The Second State of the Carbon Cycle Report (SOCCR2) estimated an net carbon sink of 154 Tg C year^−1^ for U.S. forested ecosystems (forestland remaining forestland). Our estimate for the 2001–2010 period was a net sink of 159 Tg C year^−1^, which compares well to both SOCCR2’s inventory based approach and the estimate derived from process models.

#### Net change in carbon pools

Increases in TEC were primarily the result of increases in carbon stored in live biomass (Table 1). We estimated live carbon pools increased from 16,201 Tg C to 18,890 Tg C, a net increase of 2689 (16.6%). Merchantable carbon had the largest increase in both amount (1649 Tg C) and as a percentage of 2001 stock levels (21%), followed by branches and other wood (537 Tg C; 12.5%) and coarse roots (418 Tg C; 15.5%). In total, above-ground live carbon increased by 17.3% and below-ground live carbon increased by 13.8%.

**Table 1:** Carbon stock estimates and net change in stocks for forest ecosystems of the conterminous U.S. between 2001 and 2020

	Year		Change	
Stock	2001	2020	Tg C	Percent
Aboveground live				
Biomass: Foliage	881.5	949.2	67.7	7.7
Biomass: Merchantable	7859.7	9508.9	1649.2	21.0
Biomass: Other Wood	4306.0	4842.6	536.6	12.5
Total	13047.1	15300.7	2253.5	17.3
Belowground live				
Biomass: Coarse Root	2689.4	3107.0	417.6	15.5
Biomass: Fine Root	464.6	482.3	17.7	3.8
Total	3154.1	3589.3	435.3	13.8
Deadwood				
DOM: Aboveground Medium	1488.9	1219.4	− 269.6	− 18.1
DOM: Belowground Fast	246.9	247.4	0.5	0.2
DOM: Snag Branch	238.6	281.8	43.3	18.1
DOM: Snag Stem	756.9	1028.2	271.3	35.8
Total	2731.3	2776.8	45.5	1.7
Litter				
DOM: Aboveground Fast	1382.9	1369.6	− 13.2	− 1.0
DOM: Aboveground Very Fast	856.0	904.2	48.2	5.6
Total	2238.9	2273.9	35.0	1.6
Soil				
DOM: Aboveground Slow	7140.2	6871.6	− 268.6	− 3.8
DOM: Belowground Slow	23572.1	23457.3	− 114.8	− 0.5
DOM: Belowground Very Fast	143.3	143.5	0.2	0.2
Total	30855.6	30472.4	− 383.1	− 1.2
All pools				
Total	52027.0	54413.1	2386.1	4.6

Carbon stock and net change estimates are in Tg C

The Aboveground Slow pool has been included with the IPCC Soil pool rather than the Litter pool as done in the CBM-CFS3 model

Carbon stored in dead organic matter (DOM) pools, including deadwood, litter, and soil, remained relatively stable, declining by −0.8% (−303 Tg C) over the study period. Total carbon stored in deadwood and litter increased by 1.7% and 1.6%, respectively, while soil carbon declined by −1.2%. However, the net change in DOM masked important internal dynamics (Table 1). For example, carbon stored in standing dead snags increased by 35.8% while downed deadwood (i.e., “DOM: Aboveground Medium”) declined by −18.1% with the increase in standing deadwood the result of increases in area impacted by wildfire in recent years.

**Fig. 3:**
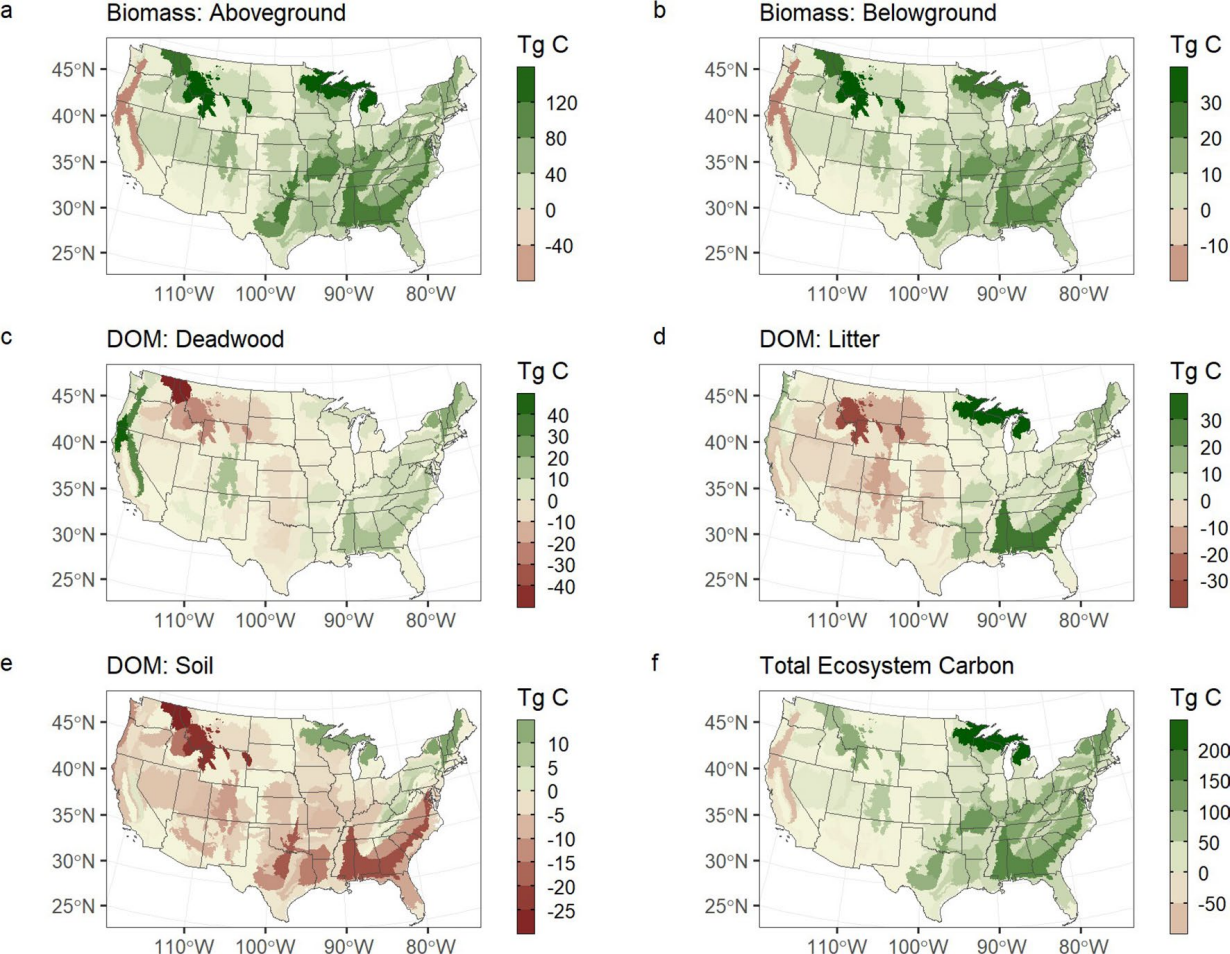
Net change in carbon pools by ecoregion for the period 2001–2020. Note, the scale for each map is different in order to highlight differences in carbon pools between regions

Net losses in live biomass carbon were concentrated in three western U.S. ecoregions (Sierra Nevada Mountains, Klamath Mountains, Cascades), primarily the result of recent high rates of wildfire. All other ecoregions in the U.S. were net sinks of live carbon (Fig. 3). The Middle Rockies and Northern Rockies ecoregions were large sinks of live carbon, resulting primarily from regrowth following high rates of historical disturbance that occurred prior to 2001. Consequently, these regions also experienced significant net declines in litter and deadwood pools as stocks decomposed over time. Deadwood pools increased significantly in western ecoregions, which have experienced several recent years of large stand-replacing wildfires. Declines in soil carbon were ubiquitous across ecoregions, although represent a relatively small portion of the overall soil pool. The largest declines were associated with near-surface soil horizons, which were most vulnerable to emissions from wildfire and increasing rates of decay due to rising temperature.

### Major controlling processes on historical net carbon fluxes

#### Sensitivity analysis

**Fig. 4:**
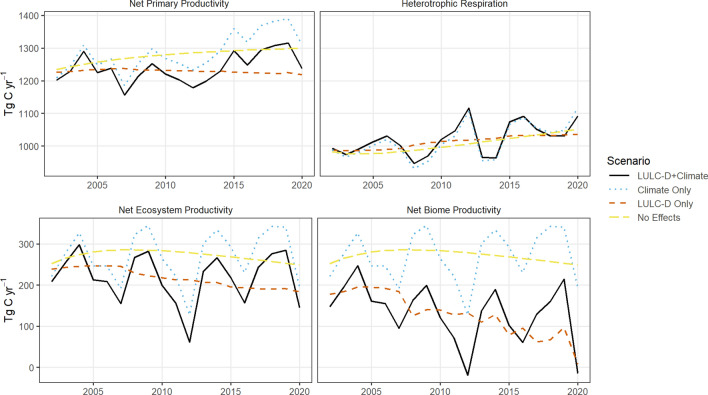
Net fluxes of carbon in conterminous U.S. forests for the period 2002–2020. The four scenarios include the effects of climate only, land use and disturbance only, the combined effects of land use and climate, and no effects other than forest aging

In addition to the reference simulation (“LULC-D+Climate”) presented in the previous section, which included the combined effects of LULC/disturbance and weather and climate, we ran three additional simulations to assess the sensitivity of major controlling processes on ecosystem carbon dynamics. The additional simulations included (a) a scenario where only weather and climate effects were included (“Climate Only”), (b) a scenario were only LULC and disturbances were included (“LULC-D Only”), and (c) a simulation where neither LULC or climate was included (“No Effects”). Figure 4 shows the estimated net primary productivity (NPP), heterotrophic respiration (Rh), net ecosystem productivity (NEP), and net biome productivity (NBP) over time for each of the four simulations.

Under the LULC-D+Climate simulation, the size of the net sink varied considerably from year-to-year, ranging from −19 Tg C year^−1^ (source) to 247 Tg C year^−1^ (sink), with climate primarily driving inter-annual variability in the size of the forest sink. When climate was not included in the simulation (“LULC-D Only”), the size of the net sink ranged from 8 to 196 Tg C year^−1^ (when 2020 is excluded, removing the effects of an extreme fire year, the low end of the range was 61 Tg C year^−1^) and showed a declining trend over time due to forest aging, increases in LULC and disturbances, and the absence of increased productivity from recent climate conditions. When LULC was excluded (“Climate Only”), the forest carbon sink was 49% higher (mean of 271 Tg C year^−1^) than simulations where LULC was included. Land use, land-use change, and disturbances were the primary controlling processes affecting the magnitude of the net carbon sink (Fig. 4).

Net primary productivity (NPP) of U.S. forests averaged 1,238 Tg C year^−1^ in the “No Effects” simulation. Incorporating the effects of LULC and disturbances reduced total NPP by 49 Tg C year^−1^, while adding the effects of climate resulted in an increase in NPP of 9 Tg C year^−1^ compared to the LULC only simulation. These results suggest that LULC and disturbance have a negative impact on NPP while climate variability and change have resulted in a small and highly uncertain increase in productivity of U.S. forests. Heterotrophic respiration (Rh) increased under all simulations resulting from increases in productivity, climate warming, and the effects of land use history. Net ecosystem productivity (NEP) (the difference between NPP and Rh) is the net carbon sink before LULC and disturbances are accounted for. Under the reference scenario (“LULC-D+Climate”), we estimated mean annual NEP at 217 Tg C year^−1^. With a mean annual NBP rate of 132 Tg C year^−1^ we attribute 85 Tg C year^−1^ to ecosystem removals resulting from LULC and disturbances.

#### Effects of LULC and disturbance

Table 2 shows the emission, harvest, and mortality fluxes for the LULC and disturbance transitions considered in this study. Reduction of carbon sequestration was primarily from the transfer of carbon to harvested wood products (64 Tg C year^−1^) with clearcut harvest accounting for more than 90% of the carbon removal from ecosystems. Harvest activities also resulted in a similar amount of carbon transferred from live to DOM pools via mortality. Our estimate of carbon transfer via harvest was considerably lower than other studies, including those from [17] (133 Tg C year^−1^), [37] (107 Tg C year^−1^), and [6] (113 Tg C year^−1^), likely the result of a large underestimation of forest thinning rates which are not well detected in the Landfire disturbance data. [38] found a similar result, where remote sensing based approaches tend to underestimate forest harvest carbon removals relative to methods, which rely on forest inventory alone.

**Table 2:** Average annual fluxes of carbon resulting from land-use and land-cover change, and disturbances for the period 2001–2020

Transition group	Transition Type	Emission	Harvest	Mortality	Transfer
Agricultural expansion	Cropland	0.58	0.44	0.26	–
	Pasture	0.56	0.49	0.25	–
	Total	1.14	0.93	0.51	–
	High Intensity	0.23	0.09	–	–
Urbanization	Medium Intensity	0.73	0.27	–	–
	Low Intensity	1.19	0.45	–	–
	Open Space	1.74	0.66	–	–
	Total	3.89	1.46	–	–
	Forest Clearcut	–	59.41	57.31	6.95
Forest harvest	Forest Selection	–	2.19	2.15	–
	Total	–	61.60	59.46	6.95
	High Severity Severity	5.60	–	8.22	–
Fire	Medium Severity Severity	3.96	–	5.68	–
	Low Severity Severity	6.34	–	6.72	–
	Total	15.90	–	20.62	–
	High Severity Severity	–	–	6.05	–
Insect	Medium Severity Severity	–	–	4.96	–
	Low Severity Severity	–	–	6.25	–
	Total	–	–	17.26	–

Emissions refer to the direct combustion of carbon to the atmosphere as either carbon monoxide, methane, or carbon dioxide. All emissions are shown in units of carbon. Harvest refers to the transfer of carbon out of the ecosystem to harvested wood products. Mortality is the transfer of live carbon to DOM pools. Transfer refers to the transfer of carbon from one DOM pool to another. Units are in Tg C year

Ecosystem carbon losses from combustion averaged 21 Tg C year^−1^, with wildfire accounting for 76% of all LULC and disturbance related emissions; emissions from urbanization and agricultural expansion accounted for 19% and 5%, respectively. Emissions from wildfire were highly variable from year-to-year, with three of the four highest fire emission years all occurring since 2017. In 2020, wildfire burned ~3.7 million hectares, primarily in California and Oregon, an amount 231% greater than the historical average. Emissions were estimated to be 73.5 Tg C (269.8 Tg CO_2_), which were more than three times the historical average.

In addition to ecosystem carbon removals, LULC and disturbances resulted in the transfer of an additional 93 Tg C year^−1^ from live to DOM pools, which will contribute to future years reductions in the carbon sink through their decay and decomposition. This future flux could be accelerated by continued increases in the turnover rate of DOM resulting from climate warming.

### Regional Case study #1: Carbon emissions from wildfire in California

Historically, wildfire in California has been an important driver of carbon balance. Over the past two decades, wildfire has taken on an increasingly important role driven in part by climate change [39, 40]. In 2020, California experienced an unprecedented amount of large high severity fires. In total, more than 4 million ha burned across the U.S. with 1.7 million ha burning in California [41] alone. Five of the six largest fires in California’s history occurred in 2020, each burning more than 100,000 ha, with the August Complex fire alone burning more than 400,000 ha across parts of five counties. Using updated fire perimeter maps [41], we were able to provide rapid assessments of impacts to carbon stocks and emissions from forested ecosystems affected by these events.

**Fig. 5:**
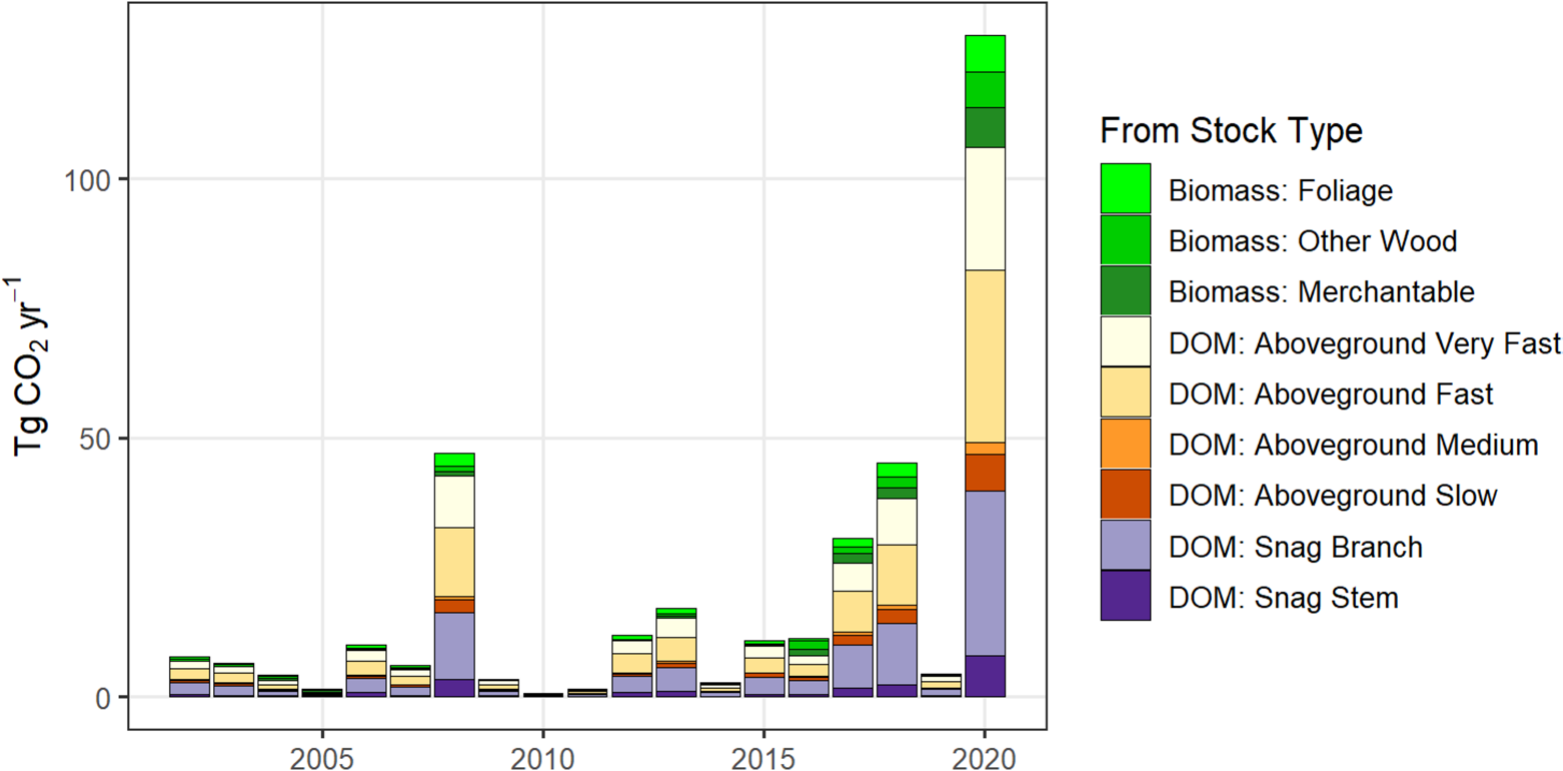
Estimates of annual wildfire emissions in California for the period 2002–2020. DOM refers to dead organic matter

Figure 5 shows the estimated annual emissions of CO_2_ in California from wildfires from each carbon pool. We estimated that between 2001 and 2010, wildfire in California resulted in 9.7 Tg CO_2_ year^−1^ being emitted to the atmosphere. Between 2011 and 2019, we estimated annual emissions from fire increased by 56% to 15.1 Tg CO_2_ year^−1^, with annual emissions exceeding 40 Tg CO_2_ year^−1^ in two of those years (2017 and 2018). These estimates compare well with estimates produced by the California Air Resources Board [42]. By comparison, the total carbon emissions from California’s commercial and residential sector averages ~43 Tg CO_2_ year^−1^ [43]. In 2020, emissions from wildfire increased 932% (relative to the 2002–2019 mean) to an estimated 127.7 Tg CO_2_ year^−1^, an amount equivalent to 75% of California’s emissions from the states entire transportation sector [43].

### Projections of carbon change under climate scenarios

We projected future changes in forest carbon dynamics for the western U.S. based on downscaled climate data for the RCP 4.5 radiative forcing scenario [44] for the period 2020–2050. We selected the CanESM2 and HADGEM2-ES365 downscaled climate futures to represent “hot-dry” and “warm-wet” futures, respectively [45]. Climate data from the Coupled Model Inter-comparison Project (CMIP5) were downscaled using the

Multivariate Adaptive Constructed Analogs (MACA) [46]. Downscaled climate data were used to produce a time series of spatial flow multipliers used to scale annual vegetation growth and the decay and decomposition of DOM at the pixel level. Future LULC and disturbances were sampled, with replacement, from historical rates of change following methods described in [31]. Additional details are described in the methods section.

Lines reflect the minimum and maximum estimate ^1^a^7^cross the two climate futures. The CanESM2 model represents the warm-wet scenario and the HadGEM2-ES model represents the hot dry scenario.

**Fig. 6:**
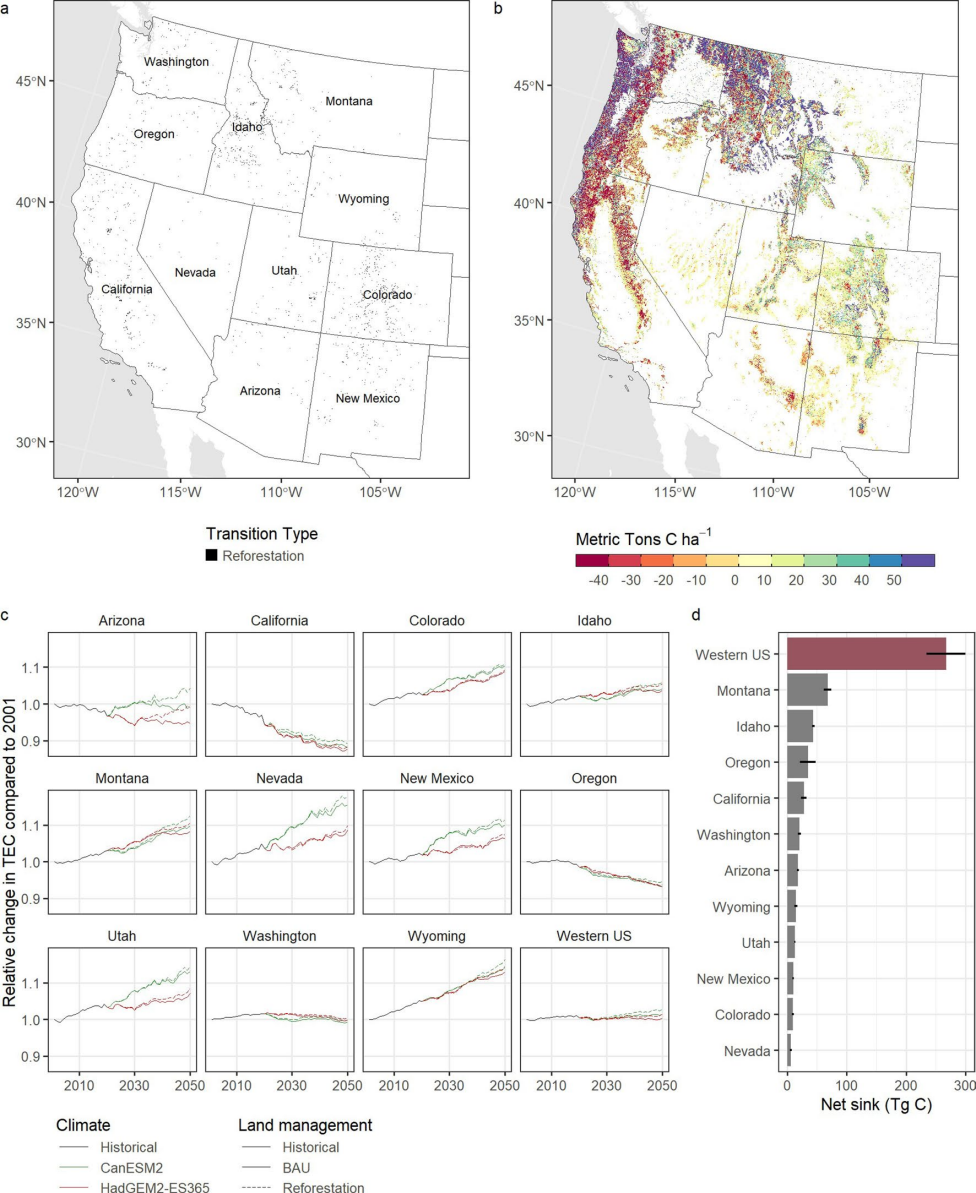
Projected change in total ecosystem carbon (TEC) for the western U.S. for the RCP 4.5 radiative forcing scenario and two climate models under both business-as-usual (BAU) and a natural climate solutions (NCS) reforestation scenario. Panel **a** shows the spatial location of areas selected for reforestation under the hot-dry scenario. Panel **b** shows the net biome productivity (NBP) between 2020 and 2050 under the hot-dry scenario. Panel **c** shows the relative change in TEC to the year 2001 for all scenarios. Panel **d** shows the mean cumulative change in NBP for the reforestation scenario relative to the BAU scenario from 2020 to 2050

Figure 6 shows the TEC storage for the historical and alternative climate futures for each of the 11 western states included in the simulation. Over the historical period, the western U.S. was a small net sink of carbon, with NBP estimated at 4.8 Tg C year^−1^. Under the warm-wet model, the size of the sink was projected to increase slightly to 6.6 Tg C year^−1^. In contrast, under the “hot-dry” model, western forested ecosystems were projected to transition to a net source of carbon to the atmosphere at an annualized rate of −0.9 Tg C year^−1^. However, the total annualized sink estimate masks considerable variability in both time and space, with some states projected as net sinks while others were projected to be a net source. While climate scenarios showed broad consistency in trajectory and magnitude of TEC for seven of the twelve western states, differences in climate scenarios are also evident. In five states, the “warm-wet” future results in greater net sequestration relative to the “hot-dry” scenario.

### Regional case study #2: Western U.S. reforestation

To demonstrate the capability of the LUCAS-CBM approach, we simulated the effects of a simple natural climate solutions (NCS) scenario where areas of grassland, shrubland, pasture, cropland, and previously burned areas that historically supported forests were candidates for reforestation. We used a set of spatial maps to constrain locations where reforestation could occur [47]. Candidate areas were identified if tree cover occurred historically and exceeded 25%. For a complete description of the methods used to identify potential reforestation sites see [47]. However, it is important to note that the methods used to generate maps of potential reforestation locations did not consider future climate conditions. It is reasonable to assume that suitability for forest restoration will be impacted by changes in climate, and therefore effect the efficacy of any mitigation strategies. Because we did not account for these effects, the example provided here should be viewed as a conceptual approach to using the modeling framework to simulate “what-if” scenarios on the effects of NCS and not a true forecast, which would imply a level of confidence not readily quantifiable given the available data.

We projected the effect of reforesting nearly 10 million ha across 11 western states beginning in 2030 and running through 2050. Reforestation of shrublands and grasslands accounted for ~75% of the total reforested area while post-fire reforestation accounted for ~17%. Reforestation of croplands and pastures accounted for less than 5% combined. When reforestation was included in the simulations, forest ecosystems sequestered an additional 234–300 Tg C by 2050 (6d). On an annualized basis, reforestation increased the annual carbon sink by 10Tg C year^−1^ under the warm-wet scenario and 7.8 Tg C year^−1^ under the hot-dry scenario.

## Discussion

The results presented above were obtained using a national approach combining the LUCAS state-and- transition simulation model of landscape change with the CBM-CFS3 model of ecosystem carbon dynamics to produce detailed, spatially explicit estimates of landscape change and ecosystem carbon dynamics for forests of the conterminous United States. The approach leverages the robust capabilities of LUCAS to represent a wide range of LULC and disturbance types derived from remote sensing techniques. We used the CBM-CFS3 model of carbon dynamics to parameterize a stock-flow sub-model and added a dynamic growth module to represent the effects of climate variability and change on the NPP of forested ecosystems. Because the method relies on the modeling of carbon fluxes to estimate stocks, the LUCAS-CBM approach provides the added benefit of providing rich detail in the underlying transfer of carbon between ecosystem pools suitable for policy-relevant applications such as national carbon monitoring, which is lacking in traditional inventory-based stock-change approaches. Additionally, because the model is parameterized at the pixel scale, all outputs are provided as a time-series of spatially explicit maps, providing much needed resolution suitable for land management decision making.

We found that U.S. forests were generally a reliable carbon sink over the past two decades, sequestering carbon at a rate equivalent to 22% of the emissions from the nations transportation sector. However, results indicate the strength of the net carbon sink declined by 38% over the last decade, resulting from forest ageing, increases in the magnitude and frequency of large natural disturbance events (e.g., drought, fire), and continued large-scale carbon removals due to harvest and urbanization. While we expect small increasing, stable, or even declining annual carbon sinks in the coming decades for western forests, large-scale reforestation presents an opportunity to reverse these trends by boosting the annual forest carbon sink strength in this region (Fig. 6). However, future climate conditions were not considered when assuming the efficacy of reforestation locations [47], resulting in large uncertainties surrounding the potential benefits of the NCS scenarios. Additional work is needed to more appropriately project the suitability of forest restoration under future climate conditions.

Results show strong agreement with a range of other studies across a number of estimated variables including estimates produced by inventory methods alone, such as the annual US EPA greenhouse gas assessment [18]. Disparate modeling approaches, from process-based to inventory-based models, are beginning to converge on a narrower range of net carbon balance for the CONUS. This study provides further evidence of the increasing stability and maturity of net carbon balance results at continental scales.

The LUCAS model was designed using the SyncroSim modeling environment, which originated as a tool to model landscape change [32]. As a result, the model contains a robust set of features to simulate a wide range of landscape change processes including vegetation dynamics [48, 49], spread and management of invasive species [50], and habitat conservation [51]. We developed the LUCAS model to account for a variety of LULC changes, including urbanization, agricultural expansion and contraction, fire, harvest, and drought mortality. However, features exist to include a range of other biophysical and socio-economic processes. For example,

[52] used the LUCAS model to track changes in water use under alternative LULC scenarios and [53] used the model to project changes in community vulnerability to coastal hazards. A range of studies have used LUCAS to assess ecosystem carbon dynamics, including a spatially explicit assessment for the state of Hawaii [54], an analysis of historical changes in the conterminous U.S. [35], and future projections for the state of California under alternative LULC and climate scenarios [31]. Ref. [55] used a high resolution version of the LUCAS framework to model carbon dynamics in a forested peatland wildlife refuge in response to repeated stand replacing fires and changes in hydrologic land management.

The linked LUCAS-CBM approach provides a middle ground between inventory-based stock-change methods and complex process-based biogeochemical models. Our method overcomes many of the limitations of inventory based estimates, notably an inability to attribute controlling processes, coarse temporal resolution, and a lack of spatially explicit estimates. Furthermore, inventory based methods often prioritize live biomass pools and in many cases under–sample other important DOM pools. We address each of these limitations while also utilizing detailed forest inventory data to parameterize key aspects of the model.

Unlike inventory-based approaches, biogeochemical models represent a wide range of underlying processes and lend themselves well to attribution of change. By design, process models are also sensitive to the effects of weather and climate variability and change [38]. However, the disadvantages of this class of models is their inherent complexity and large uncertainties resulting from the wide range of parameter estimates, which need to be made [5]. Furthermore, this class of models has been designed to work over large areas at relatively coarse resolution, which can be difficult to utilize at ecosystem management scales. The LUCAS-CBM approach was developed to overcome many of these obstacles by running on an annual time-step (as opposed to hourly or daily) and representing basic carbon transfer processes such as growth, mortality, turnover, decay, and decomposition. The relatively small number of carbon flux rates requiring parameterization still provides for attribution of change while reducing the internal complexity of the model. Furthermore, by incorporating the NPP sub-model to estimate variability in annual growth rates, our approach is sensitive to the effects of climate variability and change. Lastly, the model framework we have developed is agnostic in terms of spatial resolution, meaning it can be run at any resolution across any sized landscape provided key inputs can be obtained (e.g., downscaled climate data, LULC data) and computational resources are available.

The current implementation of the LUCAS-CBM approach utilizes a number of default parameters developed for forested ecosystems of Canada. For example, while we supplied U.S. specific merchantable growth rates based on FIA forest inventory data, we relied on the default biomass expansion factors from CBM-CFS3 to convert merchantable volume into carbon stock estimates. Additionally, we assigned each U.S. level 3 ecoregion and state to a corresponding Canadian ecozone and province so as to leverage existing volume to biomass conversion rates. For DOM, default turnover rates were based on CBM-CFS3 and then modified based on mean annual temperature for each forest type. Future research should focus on incorporating U.S. specific biomass expansion factors for individual tree species [56] and incorporating regionally specific DOM turnover rates obtained from literature. Additionally, the LUCAS-CBM approach is well suited to exploring uncertainty in carbon model parameters by drawing from statistical distributions and then sampling using Monte Carlo methods. This capability is highly conducive to exploring uncertainties in model parameters through sensitivity analysis [31, 57, 58].

The effect of CO_2_ fertilization is among the larger uncertainties associated with estimating the terrestrial carbon budget [59]. It is not currently possible to model the effects of CO_2_ enrichment on forest productivity within the CBM-CFS3 model and thus was not included in this study. However, the effects of CO_2_ enrichment could be incorporated directly within the LUCAS framework through the use of a series of temporal growth multipliers much in the same way NPP variability was modeled. This approach was used by [31] to model the effects of CO_2_ enrichment on California ecosystems under multiple climate scenarios and was shown to be a major source of uncertainty in estimating the carbon balance of terrestrial ecosystems.

The effects of LULC and disturbance are a major controlling process of ecosystem carbon dynamics. However, uncertainties in LULC can be large, depending on the underlying source of data used. Advances in remote sensing have provided a new era of land change data for modelers, yet there are significant limitations to incorporating remote sensing data into carbon assessments. Large area and high temporal frequency data describing forest disturbance, such as the North American Forest Dynamics [60] and the Global Forest Change [61] datasets, are an important contribution toward our understanding of forest disturbance dynamics. However, attribution of forest changes—i.e., harvest, fire, drought, land use—are not yet provided. As a result, we relied on the Landfire Program’s annual disturbance maps [62], which provide change attribution data. Results suggest that based on Landfire disturbance data, we underestimated the area of forest harvest, and in particular, the areal extent of selection harvest, which can be difficult to identify using synoptic-scale remote sensing data such as Landsat. [37] estimated carbon removals resulting from harvest at 107 Tg C year^−1^ with estimates ranging from 92^−1^ 45 Tg C year^−1^ across a range of other studies [20, 21, 37, 63]. These estimates are nearly two times our mean annual estimate of 64 Tg C year^−1^ based on 1.76 million ha of harvested area.

However, the harvest area for the first five years of Landfire data (2002–2007) averaged 1.12 million ha year^−1^, compared to 2.06 million ha year^−1^ for the 2008–2020 period. For this later period, total estimated carbon removals averaged 76 Tg C year^−1^. We conclude that the Landfire disturbance data (a) under-represents harvest area early in the time series and (b) systematically under-represents the amount of selection harvest overall. The effect of this data limitation would likely reduce our estimated carbon sink rate by 20–40 Tg C year^−1^ if we forced the model to achieve total carbon removals more in line with reported literature values. Reconciling these differences would greatly improve the effectiveness of any carbon monitoring program.

The generalized modeling framework also allows for additional carbon stocks and fluxes to be included, such as the lateral flux of carbon between terrestrial and aquatic ecosystems. While not included in this study, this lateral flux is considered an important factor in regional to global carbon budgets and estimated to account for ~1.0 Pg C year^−1^ globally [64] and is perhaps equal to the size of the total net terrestrial sink [65]. The LUCAS framework is also well suited to tracking the fate of carbon resulting from other lateral fluxes, such as carbon removed in harvested products. [66] used the CBM-CFS3 model linked with a harvested wood products (HWP) model that estimates emissions based on product half-life decay times to estimate carbon mitigation potential under alternative scenarios. The LUCAS framework readily allows for adding additional stock and flow pathways, which could be used to track the fate of harvested carbon (see table 2) through a range of product pools to provide a more complete understanding of forest carbon dynamics.

While we developed this study to analyze upland forested ecosystems, the framework can be extended to estimate carbon dynamics in other ecosystems as well, such as grasslands, shrublands, wetlands, agricultural lands, and urban/suburban landscapes. LUCAS has been used to model carbon dynamics in many of these systems using parameters derived from dynamic global vegetation models (DGVM’s) [35]. Future research will focus on translating those parameters into the new LUCAS-CBM framework. Additionally, researchers are using the LUCAS framework, along with the carbon stock and flow structure adapted from the CBM-CFS3 and described in this paper, to develop a spatially explicit model of ecosystem carbon dynamics in coastal herbaceous wetlands [67].

Quantifying the effects of land management actions on carbon stocks and fluxes is increasingly needed to identify opportunities for, and assess the effectiveness of, natural climate solutions (NCS). The LUCAS-CBM approach is well suited to modeling the effectiveness of a range of NCS strategies, such as changing harvest rates and geographic patterns, protecting old growth forests, reducing deforestation, and implementing reforestation programs. To date, most studies aimed at quantifying the benefits of NCS have relied on non-spatial or spatially referenced approaches, which only factor in the biophysical suitability of reforestation [7, 8] but do not consider other factors, such as areas that may provide additional co-benefits beyond increased carbon sequestration and storage [47]. A notable exception is the recent analysis of forest restoration in Canada using a spatially explicit version of the CBM approach [68]. The LUCAS-CBM approach described in this study, along with a new set of spatially explicit maps of reforestation potential [47], can be used to refine estimates of carbon sequestration benefits and assess other ecosystem service co-benefits.

## Conclusions

Previous studies have estimated temperate forests of North America as a large and persistent carbon sink over recent decades, with U.S. forests accounting for the vast majority of the continental sink. However, uncertainty in the size and variability of the sink is large, owing primarily to the wide range of methodological approaches used for estimation. Because the U.S. relies on a stock-change approach for its official reporting [18], there is a paucity of information and data on underlying carbon fluxes, making attribution of changes in the annual sink rate difficult. Additionally, while carbon stock-change estimates are updated annually, they are not spatially explicit, reducing the utility to land managers.

We developed a modeling approach to fill these important gaps. The approach described in this study was designed to serve as a middle ground between inventory-based stock-change methods and more complex process-based biogeochemical models. The LUCAS-CBM carbon monitoring and projection tool builds off a robust national forest inventory program with the added capability of producing spatially explicit carbon stocks and flows on an annual timestep. At the same time, the reduced complexity of the underlying system represented by the model makes the approach more accessible to a wider range of users. The approach is particularly well suited to producing rapid updates in response to major events (e.g., wildfire), assessing uncertainties of key model parameters (e.g., LULC change), understanding effects of key processes using “what-if” scenario analysis (e.g., climate variability, CO_2_ fertilization), or making projections over short, medium, or long time horizons. Lastly, the generalized structure of the modeling framework makes expanding the framework to cover other ecosystem types possible, as does including additional carbon flows such as lateral transfers between terrestrial and aquatic systems and harvested products.

## Methods

To estimate carbon stocks and fluxes for CONUS, we linked the CBM-CFS3 spatially referenced model of ecosystem carbon dynamics with the LUCAS spatially explicit model of LULC change. The CBM-CFS3 model was used to generate a set of forest species and ecoregion-level carbon flux rates, which were then used within the LUCAS model to produce spatially explicit maps of carbon stocks and fluxes based on LULC change, disturbances, forest aging, and climate variability and change.

### LUCAS state-and-transition simulation model

The LUCAS state-and-transition simulation model (STSM) was stratified using EPA level III ecoregions [69], U.S. states, and land management boundaries. We defined a total of 43 unique state classes which were based on the combination of classes from the National Land Cover Database [70] and the U.S. Forest Service species type-groups [71]. We defined a total of 84 transition pathways which span seven major categories of LULC change and disturbance. The model was run on an annual timestep for the period 2001–2020 at a spatial resolution of 1-km × 1-km. Below we discuss the major aspects of the LUCAS STSM and the methods used to parameterize the model. For a general description of STSM models see [32].

#### State class map

**Fig. 7:**
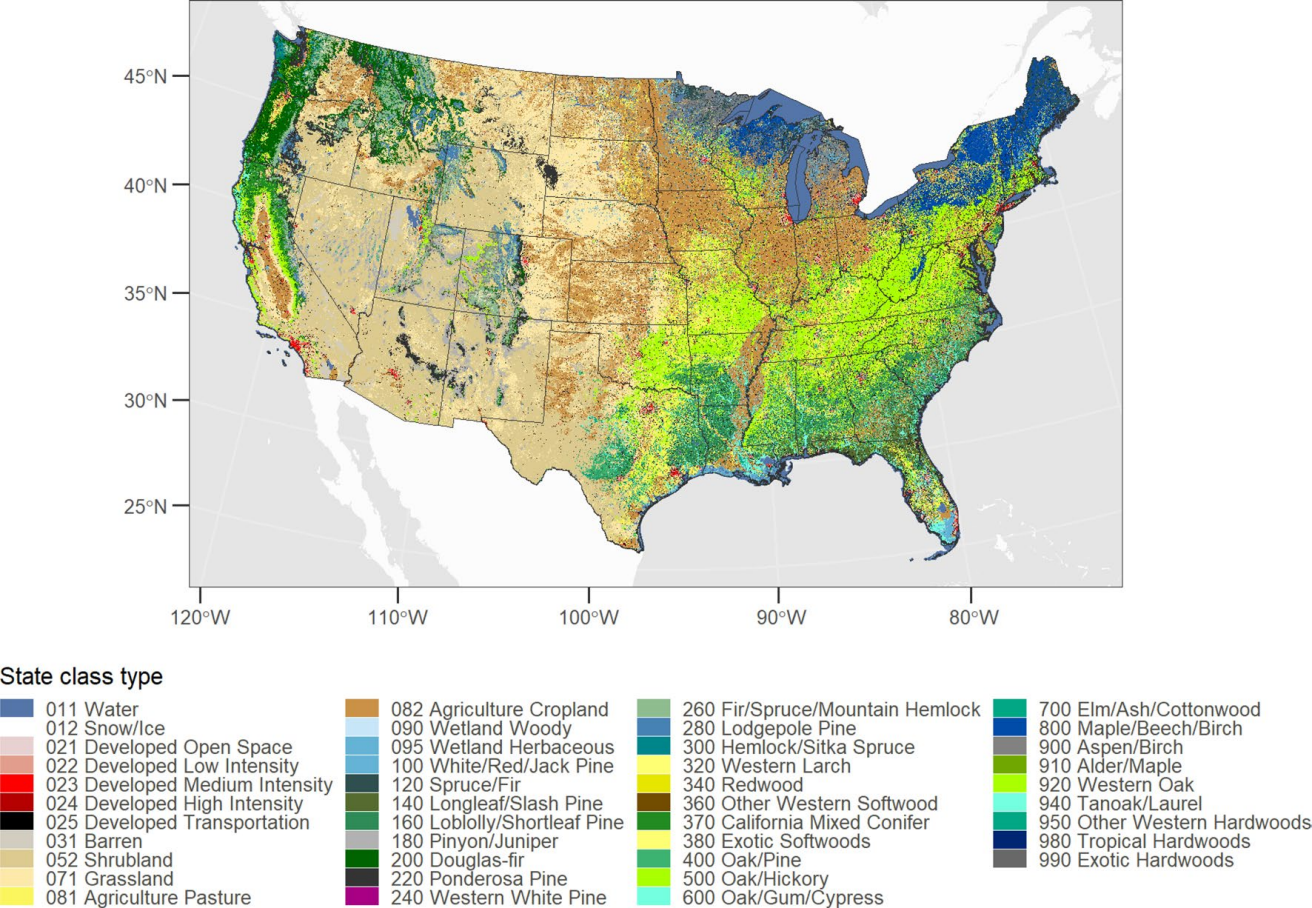
State class map developed by merging the 2001 National Land Cover Database and U.S. Forest Service forest type-groups maps

The conterminous U.S. (CONUS) was partitioned into a regular grid of 1-km × 1-km cells where each cell was assigned to a discrete land-use/land-cover classes (LULC). The initial land cover map was based on the 2001 National Land Cover Database (NLCD). We further modified the classification system to partition the three NLCD forest classes into forest type-groups based on the U.S. Forest Service (USFS) classification system. All cells mapped with a forest type-group in the USFS map were assumed to be forest cover and were recoded accordingly in our final state class map. The forest type-groups map contained 28 forest types which were generally classified as eastern and western hardwoods or softwoods. In total, the LUCAS STSM contained 43 unique LULC classes which are shown in Fig. 7.

#### Forest age

**Fig. 8:**
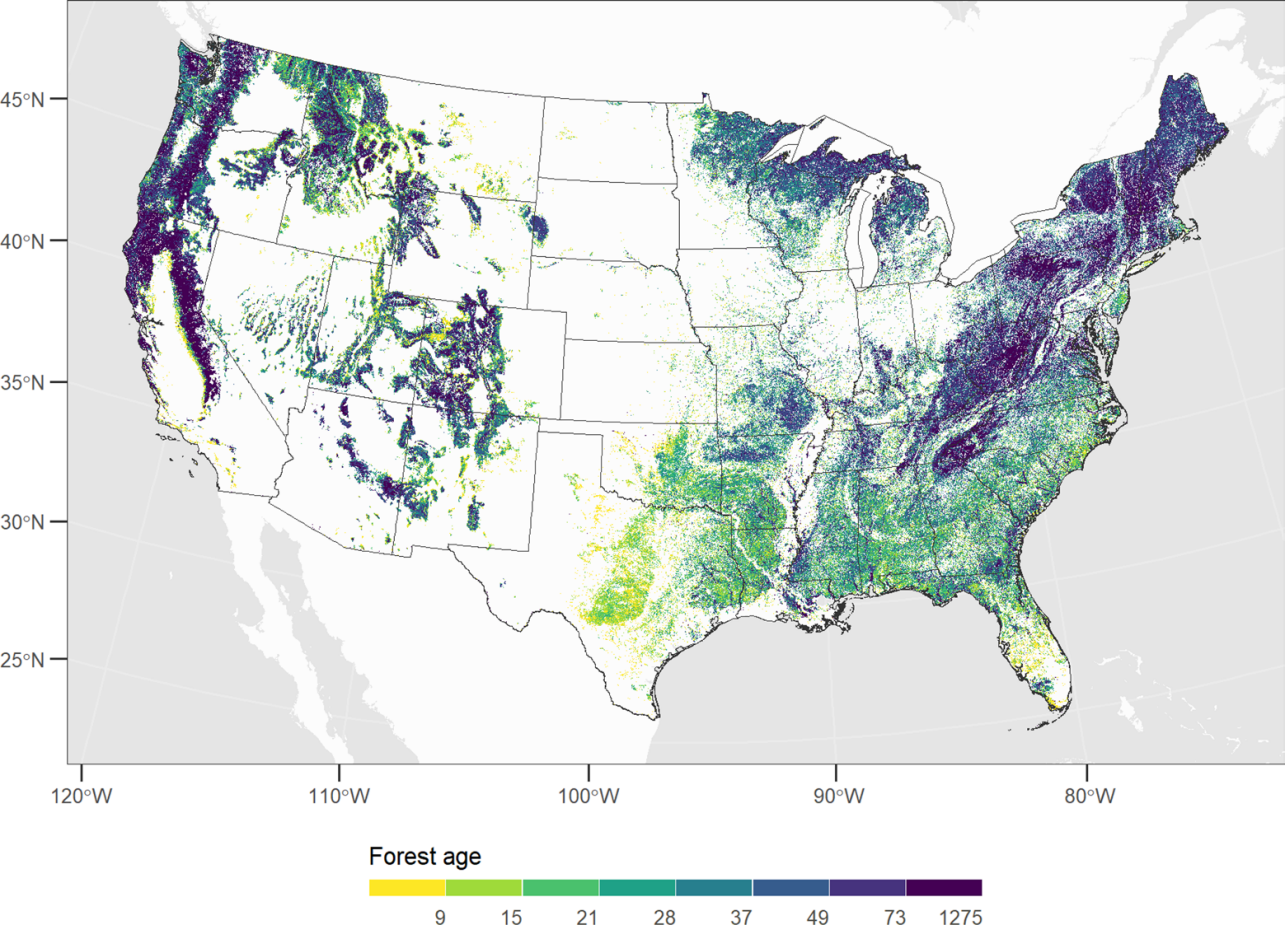
Forest age map inferred from aboveground live biomass and canopy cover data and used to initialize LUCAS model

Forest age was estimated using a spatially explicit map of aboveground live biomass [36] and a map of forest canopy cover from the National Land Cover Database. The live biomass map was used to look-up forest age for each forest type-group using the state attribute tables derived from the CBM-CFS3 reference simulations (described below). Mean canopy cover was calculated for each forest type-group and ages were scaled around the mean so as to avoid assigning low ages to forest stands with low canopy cover (Fig. 8).

#### LULC and disturbance transitions

We modeled transitions between state classes to represent major LULC change processes including: urban- ization, agricultural expansion and contraction, forest management (i.e., clear-cut and selection harvest), wildfire, and insect damage. Spatial multiplier maps, on a 1-km grid, are used to constrain probabilistic transitions and allocate deterministic transitions.

##### Land use change

For land use transitions (urbanization, agricultural expansion and contraction), we generated spatial maps of the location of changes based on the NLCD time-series maps for the years 2001, 2006, 2011, and 2016. The 5-year change rates were annualized, and the model was parameterized to stochastically select cells to transition from one class to another within the 5-year period. For timesteps after 2016, annual change rates were sampled from the historical period using a uniform distribution. For urbanization and agricultural expansion transitions, spatial multiplier maps were used to constrain the location of change by removing protected areas. For agricultural expansion, we calculated relative multipliers for each ecoregion to allocate change between cultivated croplands and the hay/pasture class with adjacency multipliers used to allocate transitions spatially. For agricultural contraction, adjacency multipliers were calculated for all forest, grassland, shrubland, and wetlands state classes and used to allocate transitions to destination classes. For urbanization transitions, adjacency parameters were used to modify the probability of change around existing developed areas. For a complete description on the methods of spatially allocating transitions see [31].

Additionally, we included changes in developed classes due to intensification (e.g., low intensity developed to high intensity developed). A time series of maps from NLCD was created showing locations where intensification occurred in each 5-year time period. The type of intensification was then estimated using relative multipliers for each intensification transition calculated for each ecoregion.

##### Forest harvest

For the forest management transitions (clearcut and selection harvest), we used annual time-series maps for the period 2001–2016 from the Landfire Disturbance database [62] to identify areas of change. For timesteps after 2016 we sampled from the historical distribution of change for each ecoregion. For clearcut cells, forest age was reset to 0; for selection harvest, the age was not reset. For both transitions we assumed cells reverted to their original state class after disturbance. In projected years, the minimum age for clearcut was based on a threshold of reaching 60% of peak merchantable volume for each forest type-group; the minimum age for selection harvest was assumed to be half the age used for clearcut harvest (see Table 3).

**Table 3:** Crosswalk table used to connect U.S. forest type-groups used in LUCAS to Ecozones, Provinces and species types from the CBM-CFS3 model

CBM_Ecozone	CBM_Province	CBM_Species	LUCAS_Species	a	b	Temp	MRI
Mixedwood Plains	Ontario	Jack pine	Forest: White/Red/Jack Pine Group	239.99	0.04	6.81	125
Atlantic Maritime	Quebec	Spruce—Genus type	Forest: Spruce/Fir Group	120.32	0.03	4.64	125
Mixedwood Plains	Ontario	Pine—Genus type	Forest: Longleaf/Slash Pine Group	148.94	0.08	19.62	125
Mixedwood Plains	Ontario	Pine—Genus type	Forest: Loblolly/Shortleaf Pine Group	225.70	0.08	17.47	125
Montane Cordillera	British Columbia	Other softwoods	Forest: Pinyon/Juniper Group	56.62	0.02	9.74	150
Pacific Maritime	British Columbia	Douglas-fir—Genus type	Forest: Douglas-fir Group	597.10	0.03	7.17	300
Montane Cordillera	British Columbia	Ponderosa pine	Forest: Ponderosa Pine Group	152.73	0.03	7.56	150
Pacific Maritime	British Columbia	Western white pine	Forest: Western White Pine Group	228.52	0.02	9.75	300
Montane Cordillera	British Columbia	Mountain hemlock	Forest: Fir/Spruce/Mountain Hemlock Group	241.10	0.03	3.80	150
Montane Cordillera	British Columbia	Lodgepole pine	Forest: Lodgepole Pine Group	203.42	0.03	3.14	150
Pacific Maritime	British Columbia	Sitka spruce	Forest: Hemlock/Sitka Spruce Group	736.44	0.04	8.38	300
Montane Cordillera	British Columbia	Western larch	Forest: Western Larch Group	298.51	0.03	5.31	150
Pacific Maritime	British Columbia	Other softwoods	Forest: Redwood Group	1907.28	0.01	12.69	1000
Montane Cordillera	British Columbia	Other softwoods	Forest: Other Western Softwood Group	138.36	0.01	2.47	150
Montane Cordillera	British Columbia	Other softwoods	Forest: California Mixed Conifer Group	477.09	0.02	10.40	150
Montane Cordillera	British Columbia	Other softwoods	Forest: Exotic Softwoods Group	363.02	0.03	7.63	150
Mixedwood Plains	Ontario	Oak	Forest: Oak/Pine Group	191.02	0.05	16.80	125
Mixedwood Plains	Ontario	Hickory	Forest: Oak/Hickory Group	206.02	0.04	13.22	125
Mixedwood Plains	Ontario	Cypress	Forest: Oak/Gum/Cypress Group	289.99	0.03	18.33	125
Mixedwood Plains	Ontario	Ash	Forest: Elm/Ash/Cottonwood Group	155.53	0.05	14.23	125
Atlantic Maritime	Quebec	Maple	Forest: Maple/Beech/Birch Group	229.04	0.03	6.71	125
Boreal Shield West	Ontario	Birch	Forest: Aspen/Birch Group	146.48	0.04	5.15	75
Pacific Maritime	British Columbia	Alder	Forest: Alder/Maple Group	436.17	0.05	10.47	300
Montane Cordillera	British Columbia	Oak	Forest: Western Oak Group	130.24	0.02	12.89	150
Pacific Maritime	British Columbia	Other hardwoods	Forest: Tanoak/Laurel Group	476.90	0.04	13.12	300
Montane Cordillera	British Columbia	Other hardwoods	Forest: Other Western Hardwoods Group	130.24	0.02	12.81	150
Mixedwood Plains	Ontario	Other hardwoods	Forest: Tropical Hardwoods Group	206.48	0.02	22.08	125
Mixedwood Plains	Ontario	Other hardwoods	Forest: Exotic Hardwoods Group	67.94	0.09	19.23	125

a and b parameters define the asymptote and rate of approach to the asymptote, respectively. Temp is the mean annual temperature across the species range; MRI is the mean historical fire return interval in years

##### Drought/insect damage

Forest mortality time series maps spanning the period 2001–2015 were used to model the effects of drought/insects and were derived from U.S. Forest Service Aerial Detection Surveys [72]. ADS data were binned into low, medium, and high severity classes based on methods described in [31]. Insect/drought disturbances were not projected beyond 2015.

##### Wildfire

To simulate wildfire we used annual fire perimeters from the National Interagency Fire Consortium (NIFC) for the period 2001–2020, which were converted into spatial multipliers in each timestep of the simulation. To estimate fire severity (high, medium, and low), we calculated severity multipliers, for each ecoregion, for all fires contained within the Monitoring Trends in Burn Severity database [73] for the period 2001–2016. Severity multipliers were then applied to the annual burn maps in each timestep and for each ecoregion, resulting in stochastic estimates of severity within each mapped fire perimeter. For grassland and shrubland state classes, all severity types were assumed to transition back into the original state class. For forest state classes, high severity fires resulted in a transition to a post-fire shrubland class, which had a 0.064 annual transition probability back into forest [31]. Medium and low severity fire reverted back into the original forest state class with no change in forest age.

### LUCAS carbon stock and flows

We adopted the carbon stock and flux structure of the CBM-CFS3 model for this study. Using the CBM-CFS3 model cross-walked to U.S.-specific forest types and parameters, we created reference simulations for carbon stocks and flows. The model structure of CBM-CFS3 was then built into LUCAS as a sub-module. The CBM-CFS3 approach includes the use of five live carbon pools (including above- and belowground pools) and 9 dead organic matter (DOM) pools covering standing dead trees, down deadwood, litter and carbon stored in soils. DOM pools are organized and named based on their rate of decay (e.g., very fast, fast, medium, slow). Carbon transfers between pools in LUCAS followed the same convention as the CBM-CFS3 model with two important modifications. First, rather than using net growth increment, we calculated net primary production (NPP) for each forest type-group and age and used this to drive annual carbon accumulation. Second, we introduced annual spatial multipliers to scale NPP based on variations in local climate conditions. These modifications are discussed in more detail below.

#### CBM-CFS3 reference simulations

We used the CBM-CFS3 model (version 1.2) to generate estimates of carbon stocks across all live and DOM pools for a 1-ha representative stand for each forest type-group. Each forest type-group was assigned to the closest forest type found in the CBM database; when no direct type was available we used generic hardwood or softwood types from CBM-CFS3. Additionally, each species was assigned to a representative ecozone and administrative boundary. We assumed wildfire was the historical stand replacing disturbance type and was the most recent disturbance while using default historical return intervals from CBM-CFS3. No additional disturbances were modeled for the reference simulation. Lastly, we calculated the mean temperature across the range of each forest type-group (Table 3), using the value to modify the CBM-CFS3 reference simulation.

##### Merchantable volume curves

The CBM-CFS3 model relies on users to provide merchantable volume curves for each tree species modeled, which are used along with a species-specific expansion factor to partition carbon into each of the five live carbon pools. We used the Von Bertalanffy growth equation to estimate merchantable volume by age. For each forest type-group, parameters used to estimate merchantable volume were queried from the U.S. Forest Service Forest Inventory and Analysis (FIA) database.

$$y = a({1} - e^{{ - b* age}} )^{3},$$

where *y* is the merchantable volume, *a* is the asymptote and *b* is the rate of approach to the asymptote (Table 3). The resulting estimates of merchantable volume by age (0–300 years), along with default biomass expansion factors from the CBM-CFS3 database, were used as inputs for the reference simulations. The CBM-CFS3 model was then run on an annual timestep for 300 years to estimate the amount of carbon stored in each pool by age by applying species-specific expansion factors to estimate biomass in each tree component. A carbon fraction was applied to estimate the carbon portion of the biomass stock. We used the default biomass expansion factors and carbon proportions from the CBM-CFS3 database.

#### Carbon flow rates: LUCAS flow pathways module

We developed a sub-module within LUCAS to calculate carbon flux parameters based on output from the reference simulations and the CBM-CFS3 database. The module uses the species crosswalk table from above, along with crosswalk tables linking carbon stocks and disturbance types used in LUCAS and CBM-CFS3. Within the LUCAS model we specified a set of carbon flow pathways defining all of the carbon pools and fluxes consistent with those used in the CBM-CFS3.

##### Net primary productivity

The module first uses results from the CBM-CFS3 reference simulation to calculate net primary productivity (NPP) as the sum of net growth and biomass turnover for a given age:

$$NPP = \sum_{a} G_{(f,b,s,fr,cr)} + \sum_{a-1} T_{(f,b,s,fr,cr)} ,
$$

where *G* is net growth, *T* is biomass turnover, *f* is foliage, *b* is branches and other wood, *s* is merchantable stems, *fr* if fine roots, *cr* is coarse roots and *a* is forest age. Thus, outputs consist of an estimate of NPP by age (up to 300 years old) and are stored as state attributes for each state class type (i.e., forest type-group). Output from the CBM-CFS3 reference simulation also allows us to calculate the proportional allocation of NPP, by age, between the five live tree component stocks.

$$pNPP_{s_i}^{a^t} = \frac{G_{s_i}^{a^t} + (S_{s_i}^{{a-1}} * T_{s_i})} {NPP^a},$$

where $pNPP_{{S_{i} }}^{{a^{t} }}$ is the proportion of NPP allocated to one of five live carbon pools ($S_{i}$) for a given aged forest (*a*^*t*^); $G_{{S_{i} }}^{{a^{t} }}$ is the net growth of carbon pool *i* at age *t*; $S_{{S_{i} }}^{{a^{t} }}$ is the amount of carbon stored in pool *i* at age *t* − 1, $T_{{S_{i} }}$ is the species and region specific carbon turnover rate, and *NPP*_*a*_ is the total species and region specific NPP for each forest age. Results are stored as flow multipliers within the LUCAS model and used to allocate annual NPP state attribute values to each live tree component.

##### Carbon flux rates

The LUCAS model uses a set of flow multipliers to estimate carbon fluxes representing biomass turnover, decay and decomposition, emissions, and transfers of carbon resulting from LULC change and disturbance. The LUCAS Flow Pathwyas module uses the set of crosswalk tables described above to parameterize a flow multipliers table for each unique combination of forest type-group and ecozone. Flow multipliers specify the annual rate and proportion of carbon transferred from one stock type to another. The ordering of carbon fluxes within the LUCAS model was established to match that of the CBM-CFS3.

1. Transfer of snag stems and branches to down deadwood pools (aboveground medium and fast, respec- tively),
2. Emission and decay of standing (snag stems and branches) and down deadwood (aboveground medium),
3. Emission from the belowground slow pool,
4. Biomass turnover from live to DOM pools,
5. Emission and decay from DOM pools (belowground very fast, belowground fast, aboveground very fast, aboveground fast),
6. Emission from aboveground slow,
7. Transfer from aboveground slow to belowground slow,
8. Growth of live pools.

In addition to the base carbon flux rates, the LUCAS Flow Pathways module also parameterizes a set of transition-based flow multiplier,s which control the rate at which carbon is transferred between pools when a change in LULC or disturbance occurs. Transition triggered flows are implemented at the end of each timestep after all base flows have occured. For this study, we considered the effects of fire (high, medium, and low severity), harvest (clearcut and selection), drought/insect mortality, urbanization, agricultural expansion (i.e., deforestation), and agricultural contraction (i.e., reforestation). Flow rates were imported from transition matricies from the CBM-CFS3 model. For wildfire, carbon flux raters were derived from [74].

#### Verification

**Fig. 9:**
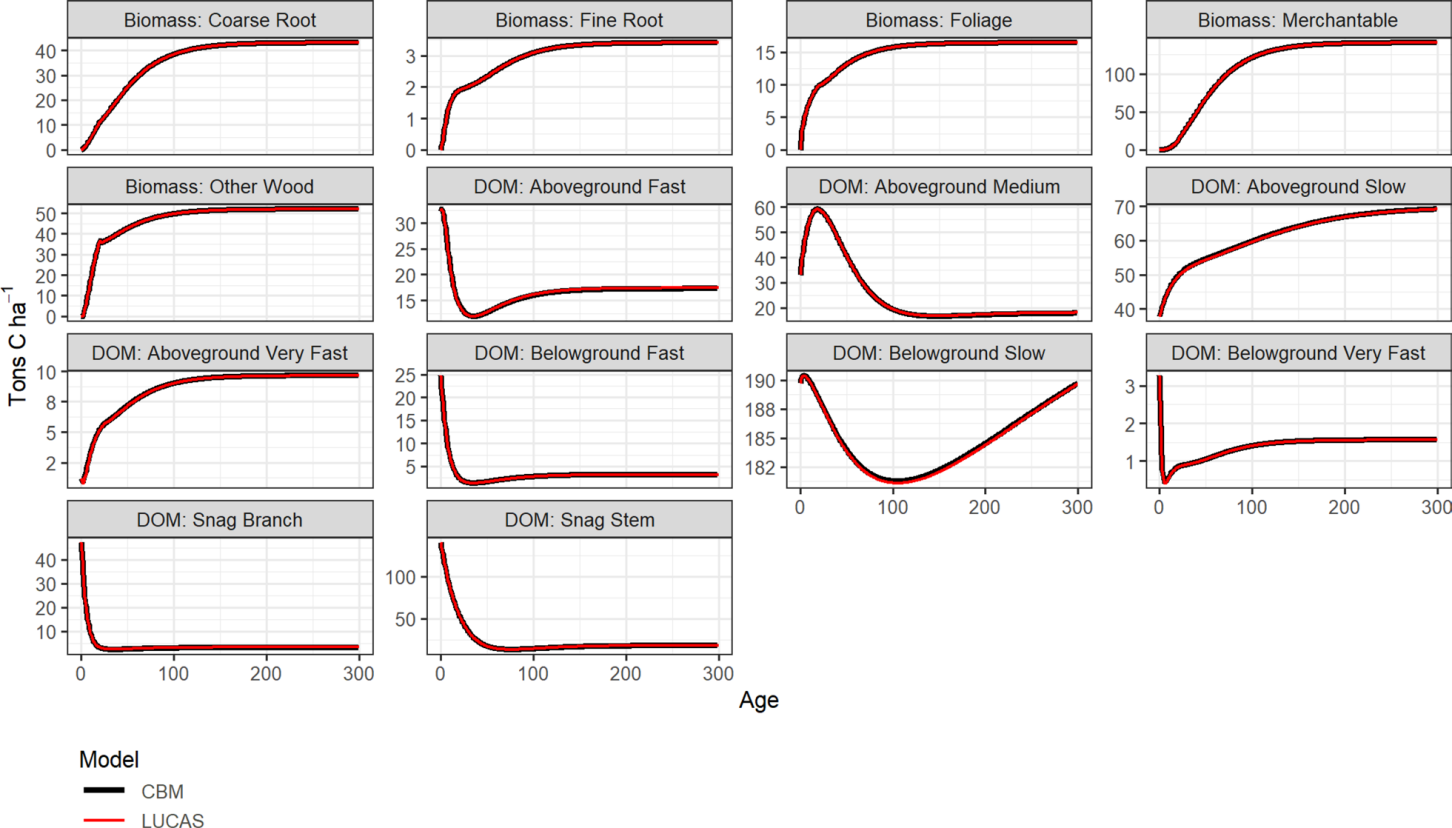
Comparison of stock estimates by age from LUCAS to output from the CBM-CFS3 model. Data shown are for the Douglas-fir forest type-group. DOM is dead organic matter

Within the LUCAS model we ran a 300 year simulation with all cells starting at age 0 using the carbon flux rates from the Flow Pathways module to estimate changes in stocks. A single 1-ha representative stand was run for each forest group-type and fire was assumed as the last stand replacing disturbance to match the assumptions from the CBM-CFS3 reference simulations. All stocks were initialized at their age-0 values obtained from the CBM-CFS3 simulations. No disturbances were simulated. We compared individual carbon stock output from the LUCAS simulation with the original output from the CBM-CFS3 reference runs to ensure we could reliably reproduce carbon stock estimates. Figure 9 shows a comparison of all stock types for the Douglas-fir forest type-group.

#### Spin-up of DOM pools

**Fig. 10:**
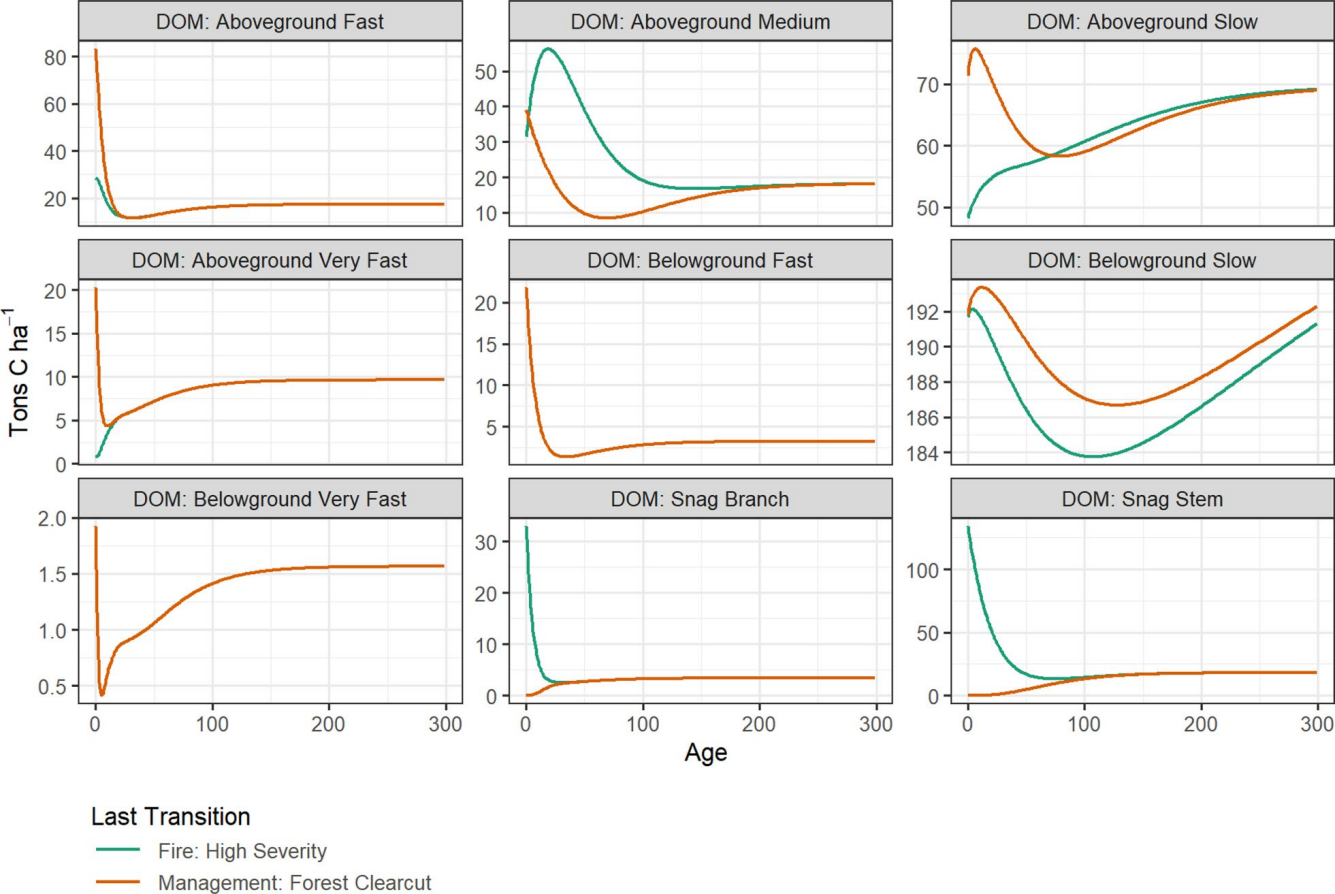
Carbon stock by age for DOM pools for cells initialized with fire or clearcut harvest as the last stand replacing disturbance. Data shown are for the Douglas-fir forest type-group. DOM is dead organic matter

The CBM-CFS3 model contains its own internal spin-up procedure for stabilizing DOM pools. However, using the CBM-CFS3 method requires running multiple iterations of the model based on the number of types of stand-replacing disturbance events considered in the model. For this reason we developed spin-up module directly with LUCAS. Within LUCAS, carbon stocks are initialized at zero and all cells are set to age zero. The historical stand replacing disturbance type was assumed to be wildfire. Flow pathways and multipliers were used to estimate carbon stocks over a 3000 year simulation period with fire events occurring based on the mean fire return interval specified for each forest type-group (Table 3). At the end of the 3000 year simulation, two types of stand replacing disturbances were simulated: high severity fire and clearcut forest harvest. After each transition, the model was run for another 300 years to generate carbon stocks by age which were used to parameterize a state attribute table in LUCAS (Fig. 10). The length of the spin-up simulation can be varied based on how long it takes DOM pools - in particular the belowground slow (soil) pool - to reach a relative steady state between successive historical disturbance events. This approach is conceptually the same as that in the CBM-CFS3, while achieving some computational efficiency when multiple stand-replacing events are considered.

#### Mapping initial carbon stocks

**Fig. 11:**
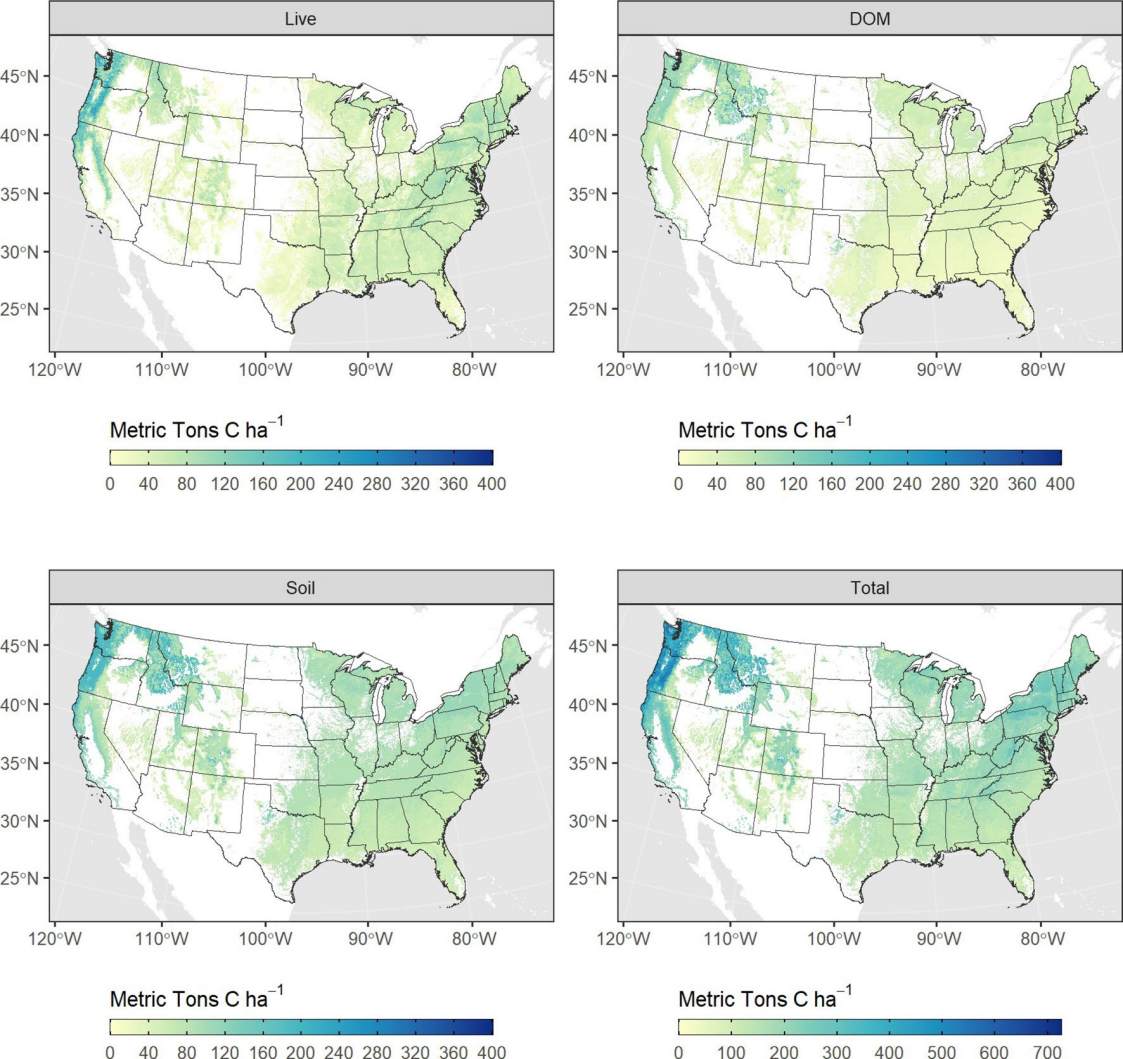
Carbon stored in live biomass, dead organic matter (DOM), and soil pools estimated for the year 2001. Also shown is total ecosystem carbon. Live carbon includes the foliage, other wood, merchantable, fine roots and coarse roots pools. DOM includes aboveground very fast, fast, medium and slow pools and belowground fast pools. Soil includes belowground very fast and slow pools. Total is the sum of all 14 live and DOM pools included in the model

To estimate spatial carbon stocks the LUCAS model was parameterized with state attribute values from the spin-up procedure described above, flow pathways and multipliers, and raster maps of forest type (Fig. 7) and and age (Fig. 8). The model was run for a single timestep for both wildfire and clearcut harvest as the last disturbance creating two sets of initial carbon stock maps. The combination of forest type and age was used to create initial stock rasters for the first timestep (Fig. 11). Fire disturbances prior to 2001 were queried from the National Interagency Fire Consortium (NIFC) and mapped to a 1-km grid. The NIFC database contains fire perimeters collected at the local level across a range of federal and state agencies and date back to the mid-1800s (the majority of records represent events from 1950-present). Cells classified in the NIFC map were assigned carbon values from the wildfire simulation; all other cells were assigned stock values from the clearcut harvest simulation.

### Spatial flow multipliers

The effects of weather and climate variability and change were incorporated into the LUCAS model to modify the annual flux of carbon in live and DOM pools. We used gridMET annual climate variables [75] to derive a set of spatial flow multipliers which were used to scale NPP and decay/decomposition of all DOM pools. For additional details on the approach see [31].

#### NPP variability

**Fig. 12:**
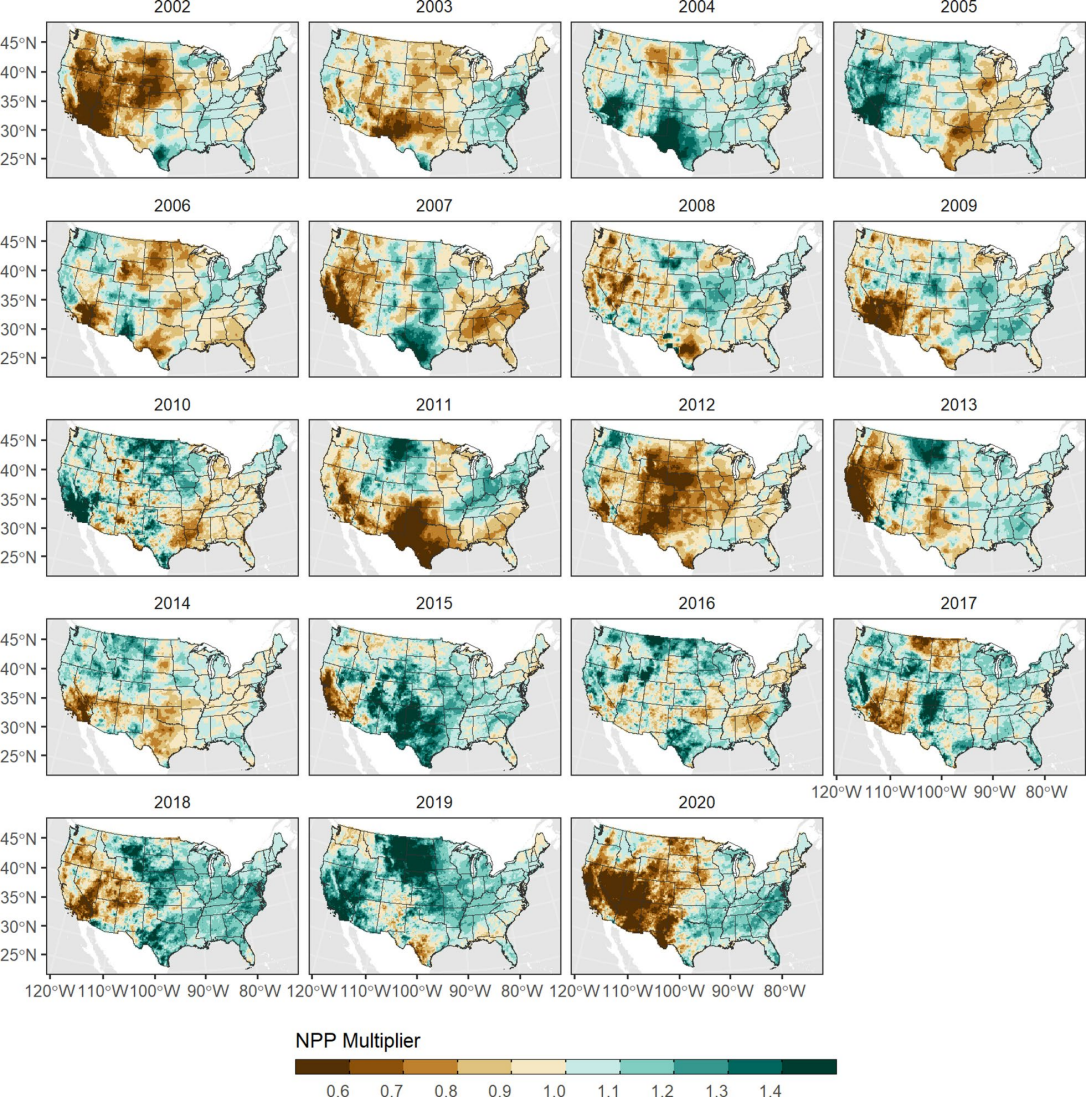
Spatial flow multipliers used to modify annual net primary productivity (NPP) in the LUCAS model

We used the NCEAS model of net primary productivity [76] to derive annual spatial multiplier maps which were used to modify the rate of vegetation growth in the LUCAS model (Fig. 12). We estimated the annual NPP multiplier using the following equations from [76]:

$$f(MAP)_t = \frac{0.551 * MAP^{1.055}} {e^{(0.000306 * MAP)}},$$

where *f* (*MAP* )_*t*_ is the NPP estimated from total annual precipitation (MAP) in a given timestep and,

$$f(MAT)_t = \frac{2540} {1 + e^{(1.584 - 0.0622 * MAT)}},$$

where *f* (*MAT* )_*t*_ is NPP estimated from total mean annual temperature (MAT) in the same timestep. Annual NPP was then estimated as:

$$NPP_t = MIN[f(MAP)_t, f(MAT)_t].$$

We also applied the same equations to gridMet climate normals for the period 1980–2010 (*µN PP*_1980−2010_). The annual NPP multiplier for each cell was estimated as:

$$NPP_{anom} = \frac{NPP_t} {\mu{NPP}_{1980-2010}}$$

and applied to the age and forest type specific NPP estimates derived from the CBM-CFS3.

#### Decay and decomposition of DOM

**Fig. 13:**
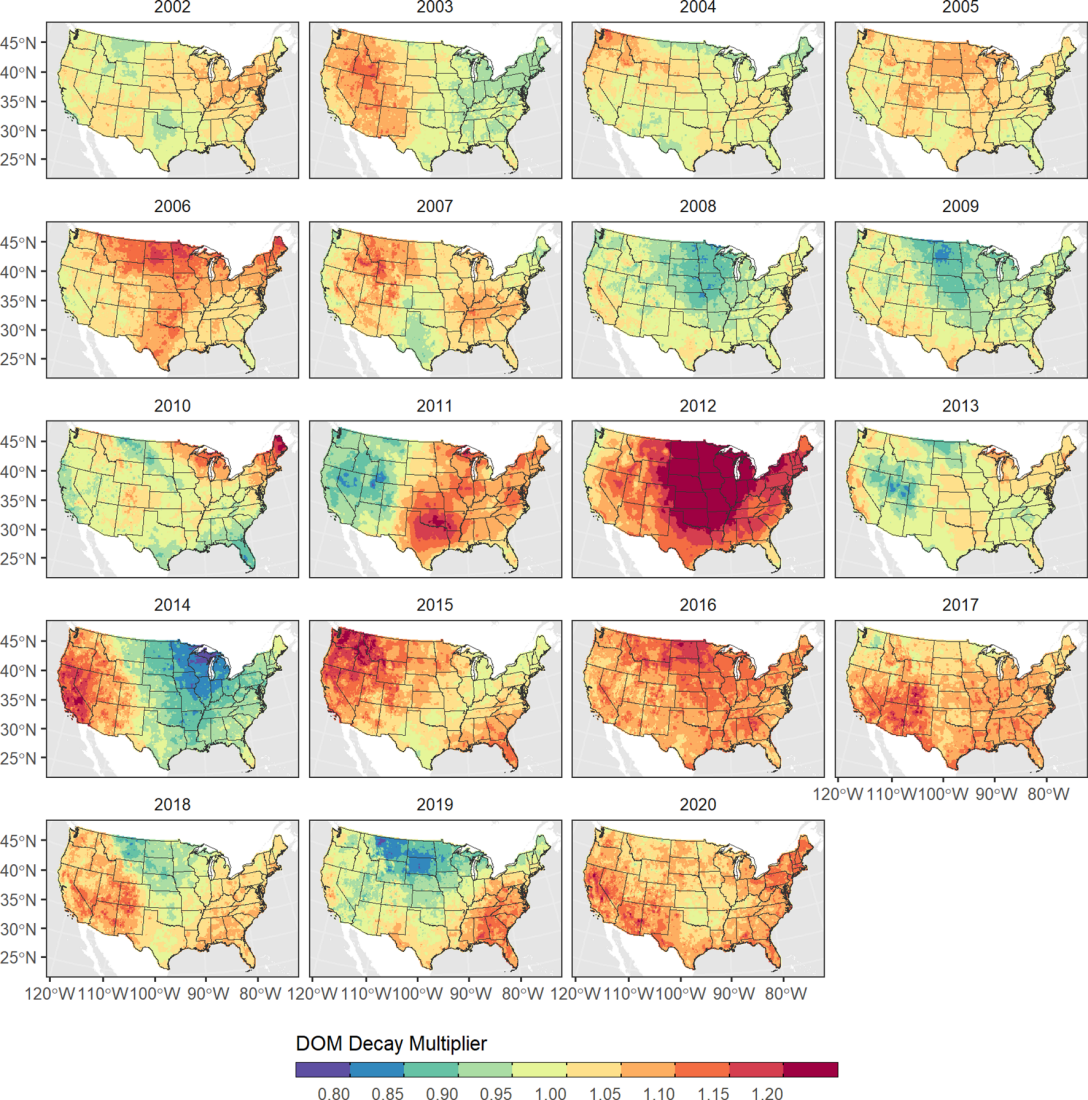
Spatial flow multipliers used to modify annual decay of dead organic matter (DOM) in the LUCAS model

Decay of DOM pools was modeled using a temperature-dependent decay rate for each DOM pool based on methods from [11]. Mean annual temperature for each species was used to scale base decay rates (*BDR*) of DOM for a 10 °Creference temperature (see Table 3). The effective decay rate (*EDR*) for each DOM pool and species type-group was calculated using Q10 coefficients for fast (2.65) and slow (2.00) DOM pools as:

$$EDR = BDR * e^{(T-T_{ref}) * ln(Q_{10}) * 0.1}$$

where *T* is the mean annual temperature across a species range and *T*_*ref*_ is the 10 °C reference temperature. To represent spatial and temporal variability in decay of DOM we calculated a time-series of annual maps, which scale the effective decay rate at the pixel scale using two *Q*_10_ coefficients. A *Q*_10_ rate of 2.65 was used for Aboveground Very Fast and Aboveground Slow pools while a *Q*_10_ of 2.0 was used for all other DOM pools. Notably, the Q10 value of 2.0 was also applied to the below-ground slow (soil) pool, whereas the default rate from CBM-CFS3 uses a *Q*_10_ of 1.0. We chose this modification based on recent studies suggesting a stronger effect of climate warming on the decomposition of soil pools. For example, [77] suggested a *Q*_10_ rate of 1.23 based on a study of Canadian forests while [78] and [79] suggested a *Q*_10_ of 2.7 and 2.3, respectively, based on a soil warming study in a western U.S. coniferous forest. Thus, the modified decay rate (*MDR*) for a cell *c* in timestep *t* was calculated as:

$$MDR_{c,t} = EDR * DM_{c,t}$$

where *DM* is the decay multiplier in cell *c* in timestep *t*. The annual spatial decay multiplier was calculated as:

$$DM_{c,t} = 1*Q_{10}^{(T_{mean}-T_{norm})/10)}$$

where *T*_*mean*_ is the mean annual temperature in timestep *t* and *T*_*norm*_ is the mean annual temperature for the 30-year climate normal. Figure 13 shows the DOM decay spatial multipliers for used for the fast pools.

### Scenario simulations

All historical scenario simulations were run on an annual timestep for the period 2001–2020 at 1-km × 1-km spatial resolution. The primary simulation included the combined effects of LULC change, ecosystem disturbance, and climate change on the historical carbon balance of forest ecosystems in the conterminous United States. To better understand the major controlling processes of historical carbon dynamics, we also ran three additional simulations where (1) no LULC or disturbance was simulated, (2) no climate variability was simulated, and (3) no climate or LULC effects were simulated. Outputs from the simulations are available as both spatial and tabular files. Spatial outputs include annual carbon stocks and fluxes, LULC composition (i.e., state class maps and forest age), and LULC transitions.

For the western U.S., we ran four simulations which project carbon balance out to 2050 under the RCP 4.5 radiative forcing scenario. We used two climate models representations of future conditions to represent “hot-dry” (HadGEM2-ES365) and “warm-wet” (CanESM2) futures [45]. For each of the two climate models we also ran a reforestation scenario where ~10 million ha of non-forest lands were converted to forest beginning in 2030 based on a map of lands that historically supported forests [47]. Downscaled climate data from the Multivariate Adaptive Constructed Analogs (MACA) [46] database were used to generate an annual time series of spatial growth multipliers and DOM decay multiplies using the method described above. Future land use was modeled by bootstrap sampling each transition type from the full historical record following methods described in [31].

All simulations were run on a desktop workstation running Windows 10 with 24 cores and 256 Gb of RAM. Simulations were run using SyncroSim version 2.2.27, and package versions 3.2.28 (stsim), 3.2.17 (stsimsf), and 1.0.7 (stsimcbmcfs3).

## Data Availability

All output tables and time series of raster maps are available on the U.S. Geological Survey’s ScienceBase data repository at 10.5066/P9QUIRNP. A fully functioning LUCAS model with all scenarios included in this study is available on the lead authors Github repository (https://github.com/bsleeter/lucas-national-assessment) along with the manuscript, all supporting data noted in text, figures, and tables.
